# Dynamic alterations in the donkey fecal bacteria community and metabolome characteristics during gestation

**DOI:** 10.3389/fmicb.2022.927561

**Published:** 2022-08-18

**Authors:** Zhenwei Zhang, Bingjian Huang, Yonghui Wang, Yandong Zhan, Mingxia Zhu, Changfa Wang

**Affiliations:** Liaocheng Research Institute of Donkey High-Efficiency Breeding and Ecological Feeding, Agricultural Science and Engineering School, Liaocheng University, Liaocheng, China

**Keywords:** donkey, fecal bacteria, plasma, metabolome, gestation

## Abstract

In donkeys, the gestation period is a dynamic and precisely coordinated process involving systemic and local alterations. Both the gut microbiota and its link with blood metabolites are thought to play significant roles in maintaining maternal health and supporting fetal development during the gestation period. This study was conducted to evaluate gut microbiota changes and the correlation with plasma metabolites in Dezhou donkeys during the gestation period. The donkeys were divided into the four following groups according to their pregnancy stages: the non-pregnant (NP), early stage of pregnancy (P1), middle stage of pregnancy (P2), and late stage of pregnancy (P3) groups. A total of 24 (*n* = 6 per group) samples of donkey feces and plasma were collected. The results showed that the diversity (Shannon index) of fecal bacteria significantly increased throughout the gestation period. The phyla Spirochaetota and Fibrobacterota varied significantly according to the stages of pregnancy (*p* < 0.05). At the genus level, the abundance of *Treponema* in pregnant donkeys was greater than that in non-pregnant donkeys (*p* < 0.05), and the genus *Streptococcus* reached its maximum abundance in the P2 period (*p* < 0.05). The abundance of *Ruminococcaceae*_NK4A214_group and norank_f_norank_o_WCHB1-41 linearly increased with the progression of pregnancy (*p* < 0.05). In addition, the host plasma metabolome was altered significantly during the gestation period. Testolic acid, estradiol-17beta 3-sulfate, equol 7’-o-glucuronide, equol 4’-o-glucuronide, estrone, estrone 3-glucuronide, and estradiol were the most significant differential enriched metabolites, and they increased gradually as gestation progressed. The altered metabolites were mainly enriched in pathways matched to bile secretion, ABC transporters, amino acid metabolism, protein digestion and absorption, mineral absorption, fatty acid degradation, glycerophospholipid metabolism, and steroid hormone biosynthesis. We also found a significant correlation between the shifts in donkey fecal bacteria and changes in the host metabolism. In summary, this study provided systematic data on the fecal bacterial changes and host plasma metabolism of donkeys throughout pregnancy. The results indicated that host–bacteria interactions during the gestation period influence the host metabolism. These interactions benefit the pregnant donkeys by providing a sufficient supply of nutrients and energy for fetal growth and maternal health.

## Introduction

In China, in ancient times, the donkey was used for farm tillage, pulling carts, pack transport, and milling (Martin-Rosset, 2018). Nowadays, with the development of rural districts and the mechanization of agriculture, the modern donkey industry mainly focuses on the utilization of fur, meat, and milk (Zhang et al., 2021). The new interest and increasing demand for donkey products have stimulated the rapid advancement of intensive feeding and the breeding of donkey.

The donkey belongs to the Equus family, and it has a specific physiology and metabolism. It is a territorial, non-seasonal, polyestrous, and single-bearing breeder. The gestation length for donkeys ranges between 333 and 395 days, with an average of 371 days (Miragaya et al., 2018). Donkeys’ gestation length of donkeys is not affected by the year of foaling, the birthweight of the foal, or the age of the jenny (Miragaya et al., 2018); however, the lengthy gestation period usually causes the generally low reproductive efficiency of donkeys.

The gestation period is a dynamic and precisely coordinated process involving systemic and local changes in jennies that support the supply of nutrients to the conceptus for growth *in utero* (Napso et al., 2018). The prenatal period represents a unique physiological paradigm and is of critical importance for donkeys. However, information regarding the gestational physiology of the jenny is limited. Generally, pregnancy is characterized by dramatic variations in hormones, metabolism, and immune functions (Motosko et al., 2017). The analysis of plasma has been accepted to identify typical compounds to assess the underlying metabolic changes that occur during the gestation period (Cheng et al., 2018). Until now, however, no study has been conducted to investigate blood metabolites in donkeys throughout pregnancy.

Evidence shows that pregnancy is related to metabolic changes that may be associated with microbiota compositional dynamics (Huang et al., 2019; Ji et al., 2019). Several physiological adaptations occur during pregnancy to maintain maternal health and support fetal development (Motosko et al., 2017). Together with the physiological adaptations of gestation, changes in microbiome composition are observed as pregnancy progresses (Franks, 2012; Cheng et al., 2018; Li et al., 2022). Koren et al. (2012) reported that in humans, the composition of the gut microbiome during the first trimester of pregnancy is quite similar to that observed in non-pregnant women. Yet, the microbial diversity increases while richness decreases with pregnancy progression, and Proteobacteria and Actinobacteria show enriched abundance in the third trimester. However, Jost et al. (2014) and DiGiulio et al. (2015) reported few modifications to the taxonomic composition and diversity of the microbiota composition during pregnancy. Thus, the exact effect of gut microbiota and the balance of these microorganisms on the complex process of sustaining fetal development and maintaining maternal health during pregnancy is not yet known (Nelson et al., 2016).

As herbivores, donkeys utilize feedstuffs via microorganism fermentation in the hindgut (Zhang et al., 2022), and bacteria are the primary degraders of plant fiber in the donkey hindgut (Santos et al., 2011). They are the most abundant and metabolically active microorganisms, enabling donkeys to ferment plant material to generate volatile fatty acids (VFAs; Liu G. Q. et al., 2019). Therefore, the bacteria in the donkey hindgut constitute a complex ecosystem involving a symbiotic relationship with the host that plays an important role in nutrient digestion, immune programming, pathogen prevention, and metabolic status (Santos et al., 2011). Gut bacteria also exhibit a vast array of complex metabolic activities that synthesize various compounds, including VFAs, ammonia, bioamines, vitamins, and lipid compounds (Blachier et al., 2017). After being absorbed into the blood, these compounds can be modified by the host and may be actively involved in the host cells as co-metabolites.

Recently, the roles of gut bacterial activity in regulating host physiological functions in association with metabolic changes during pregnancy have attracted considerable interest in humans, sows, and ruminants (Koren et al., 2012; Liu H. B. et al., 2019; Wang et al., 2021). However, few studies have explored the shifts in fecal bacteria and corresponding changes in plasma metabolites in donkeys throughout pregnancy. Therefore, this study was conducted to evaluate such factors in the context of donkeys. We hypothesized that donkeys fed a diet meeting the NRC recommendations during the entire gestation period would experience bacterial composition and plasma metabolite changes in the hindgut. The bacterial composition of feces and the metabolite composition of plasma in donkeys were determined on days 0, 150, 210, and 270 of pregnancy. The results reported here have important implications for understanding the relationship between gut microbiota and pregnancy.

## Materials and methods

### Animals

Twenty-four jennies (aged 5 years) belonging to the Dezhou donkey breed were enrolled in the study. After mating, donkeys were housed in collective paddocks (30 animals/each, 20 × 20 m). The diet was based on wheat straw *ad libitum* along with a commercial concentrate feed formulated for the gestation period (Hekangyuan Co., Ltd., Shandong, China) according to the nutrient requirements proposed by the National Research Council Donkey and other equids (2007) recommendations. The composition and nutrient levels of the basal diet for Dezhou donkeys were shown in Supplementary Table 1. Donkeys were administered twice daily (09:00 and 17:00) at 1.5% of their body weight. All donkeys were healthy, with no history of any disease, and they had free access to water throughout the experiment. In addition, none of the donkeys had been administered medicine or antibiotics within the past 6 months.

### Sampling

The donkeys mated naturally and were diagnosed with pregnancy via B-ultrasound examination at 60 days. The donkeys were divided into four groups according to their pregnancy stages: the non-pregnant (NP, before mating), early stage of pregnancy (P1, fifth month after mating), middle stage of pregnancy (P2, seventh month after mating), and late stage of pregnancy (P3, ninth month after mating) groups. A total of 24 fresh fecal samples and 24 plasma samples were obtained from the four groups. Fresh fecal samples of each animal were collected before morning feeding via grab sampling through rectal palpation. The fecal samples (2 g each) were immediately transferred into sterilized tubes and stored in liquid nitrogen at –80°C for DNA extraction. Blood samples were collected from the jugular vein of the donkeys after 2 h of morning feeding and transferred into a vacuum tube containing EDTA. Plasma was obtained by centrifugation at 4°C for 10 min at 3,000 × *g* and stored at –80°C.

### DNA extraction, polymerase chain reaction amplification, and Illumina MiSeq sequencing

Total genomic DNA of each feces sample was extracted using the E.Z.N.A.^®^ Soil DNA Kit (Omega Bio-tek, Norcross, GA, United States) according to the manufacturer’s instructions. The concentration and purity of the microbial DNA was determined using a NanoDrop 2000 UV-vis spectrophotometer (Thermo Scientific, Wilmington, DE, United States), and the quality of DNA was evaluated using 1% agarose gel.

The 338F (3’-ACTC CTAC GGGA GGCA GCAG-5’) and 806R (3’-GGAC TACH VGGG TWTC TAAT-5’) primers were used to amplify the hypervariable regions of the 16S rRNA V3-V4 gene using the ABI GeneAmp^®^ 9700 PCR thermocycler (ABI, CA, United States). Polymerase chain reaction (PCR) was conducted in triplicate with the TransStart Fastpfu DNA polymerase in a total reaction volume of 20 μL containing 2 μL of 2.5 mM dNTPs, 4 μL of 5 × FastPfu buffer, 0.8 μL of forward and reverse primers (5 μM), 0.2 μL of bovine serum albumin, 0.4 μL of FastPfu polymerase, 10 ng of template DNA, and ddH_2_O. Then, the amplicons were detected by electrophoresis on a 2% agarose gel and further purified via an AxyPrep DNA Gel Extraction Kit (Axygen Biosciences, Union City, CA, United States) and quantified with Quantus Fluorometer (Promega, United States). Purified amplicons were pooled together in equimolar concentrations and paired-end sequenced (2 × 300 bp) using an Illumina MiSeq platform (Illumina, San Diego, United States) at Majorbio Bio-Pharm Technology Co. Ltd. (Shanghai, China) with the standard protocols.

### Sequence processing and bioinformatics analysis

Raw data with FASTQ files were quality filtered by Trimmomatic and merged via FLASH according to the criteria described by Liu C. et al. (2019). To minimize the effects of sequencing depth on microbial diversity and composition determination, the number of reads from each sample was rarefied to 43,080. The QIIME (version 1.9.1) and UPARSE (version 7.1) were applied for analysis of the sequences and clusters of the operational taxonomic units (OTUs) with a 97% similarity cutoff. Meanwhile, the taxonomy of each OTU representative sequence was analyzed using the Ribosomal Database Project (RDP) Classifier algorithm against the Silva (SSU138) 16S_bacteria database by a confidence threshold of 0.7 (Liu C. et al., 2019).

Bioinformatics analysis was carried out on the free online site of the Majorbio I-Sanger Cloud Platform.^1^ The alpha diversity indexes, including the ACE, Chao, Sobs, and Shannon indexes, as well as Good’s coverage, were calculated using MOTHUR (version v.1.30.2) at a sequence depth of 29,933 for all samples. A rarefaction curve, Venn diagram, and bar graphs were obtained using the R language (version 3.3.1). Beta diversity was estimated by calculating the weighted normalized UniFrac distance (phylogenetic information and abundance to compute community similarity) and visualized by principal coordinates analysis (PCoA). The PCoA plots of the dissimilarity metrics were produced using the ape packages in R (version 3.3.1). Partial least squares discriminant analysis (PLS-DA) was performed to visualize the bacterial alterations among experimental groups, and the results were plotted using the mixOmics and plsda packages in R (version 3.3.1). The Kruskal–Wallis H test was used to determine the phyla and genera that presented significant differences in abundance among groups with the stats package in R (version 3.3.1) and the scipy package in PYTHON. The linear discriminant analysis effect size (LEfSe) was applied to detect bacterial biomarkers during different gestation periods based on *p*-values < 0.05 and LDA scores > 3.5. Potential function capacities were predicted on PICRUSt (1.0.0) software using 16S rRNA sequencing data (Huang et al., 2019).

### Liquid chromatography–mass spectrometry metabolomics profiling

A total of 24 plasma samples were thawed on ice and assessed using a liquid chromatography–mass spectrometry (LC-MS) platform (UHPLC-Q Exactive HF-X system, Thermo Fisher Scientific Inc., MA, United States). During the analysis, quality control (QC) samples prepared by mixing all plasma extraction aliquots were set to monitor the stability of the analysis.

The 100 μL of each sample was mixed with 400 μL of precooled acetonitrile and methanol (1:1, v/v) with 10 μL of internal standard (0.02 mg/mL, L-o-chlorophenylalanine) in the centrifuge tubes. Mixed samples were then vortexed for 30 s and settled at –20°C for 30 min. After being centrifuged at 4°C for 15 min at 13,000 × *g*, 200 μL of the supernatant was collected and transferred to a clean vial for LC-MS/MS analysis.

The treated sample (2 μL) was injected into a 100 × 2.1 mm ACQUITY UPLC HSS T3 column (Waters, Milford, United States) packed with 1.8 μm particles and preheated at 40°C for chromatographic separation of plasma metabolites. MS data were obtained using a Thermo UHPLC-Q Exactive Mass Spectrometer (AB Sciex, United States) equipped with an electrospray ionization (ESI) source operating in either positive (ESI +) or negative (ESI-) ion mode. In ESI + mode (basic species), samples were retained and gradient eluted from the column using solvent A composed of 95% water and 5% acetonitrile with 0.1% formic acid. In ESI- (acidic species), samples were eluted from the column using solvent B composed of 5% water, 47.5% acetonitrile, and 47.5% isopropanol with 0.1% formic acid. The elution process was performed at a flow rate of 0.4 mL/min, and the column temperature was maintained at 40°C. The solvent gradient (A:B) changed according to the following conditions to equilibrate the systems: from 0 to 3.5 min, 100%:0% to 75.5%:24.5%; from 3.5 to 5 min, 75.5%:24.5% to 35%:65%; from 5 to 7.4 min, 35%:65% to 0%:100%; from 7.4 to 7.6 min, 0%:100% to 48.5%:51.5%, from 7.6 to 7.8 min, 48.5%:51.5% to 100%:0%; and from 7.8 to 10 min, 100%:0% to 100%:0%. The optimal heat and capillary temperatures were 425°C and 25°C, respectively. The mass scanning was set to a range of 70–1,050 m/z. The flow rates of sheath gas and aux gas were 50 arb and 13 arb, respectively. Both the ESI + and ESI- ion-spray voltages were 3.0 kV. Data acquisition was performed with the Data Dependent Acquisition (DDA) mode.

### Metabolic data preprocessing and annotation

Raw data from the UPLC-time of flight (TOF)/MS were imported into the Progenesis QI 2.3 (Non-linear Dynamics, Waters, United States) for peak detection, alignment, and peak filtering. The preprocessing results produced a data matrix consisting of the mass-to-charge ratio (m/z) values, retention time (RT), and peak intensity. Each retained peak was then normalized using MetNormalize. The relative standard deviation (RSD) value of the metabolic features in the QC samples was set at a threshold of 30% to standardize the reproducibility of the metabolomic data.

For the annotation of plasma metabolic features, the Human Metabolome Database (HMDB) and Metlin database were applied to align the m/z data and identify relevant metabolites. The mass tolerance between the measured and theoretical masses of the components of interest was 10 ppm. The confidently identified metabolites were further validated by the MS/MS fragments score (> 30) and isotopic distribution measurements.

### Multivariate statistical analysis of metabolomic data

The multivariate statistical analysis for plasma metabolites was conducted using the ropls package (version 1.6.2) in R on the Majorbio I-Sanger Cloud Platform. The positive and negative data were obtained and analyzed using the SIMCA-236 P software package. After the metabolite variables were scaled to unit-variances, principal component analysis (PCA) was performed to visualize the metabolic differences among experimental groups using an unsupervised method. After the metabolite variables were scaled to Pareto scaling, orthogonal PLS-DA (OPLS-DA) was applied for statistical analysis to visualize the global metabolic alterations among comparable groups. To avoid the risk of overfitting, the model parameters R2 and Q2 were computed to evaluate the interpretability and predictability of the models. Variable importance in the projection (VIP) was calculated in the OPLS-DA model to present the overall contribution of each variable. If the VIP > 1.0, the variables are considered relevant for group discrimination. The *p*-values were estimated on one-dimensional statistical analysis with the paired Student’s *t*-test, and differences were declared at *p* < 0.05.

### Differential metabolic features and correlation analysis

One-way analysis of variance (ANOVA) tests were conducted to identify the statistically significant metabolites among the groups at a significant threshold of FDR < 0.05. A total of 11,836 differential peaks were identified, including 5,312 peaks in the ESI + and 6,524 peaks in the ESI- model. Differential metabolites among the four groups were summarized and matched to the KEGG database using untargeted metabolomic pathway analysis and metabolic enrichment. Screened differential metabolites were analyzed for expression pattern clustering using the gplots package in R. The distance calculation algorithms mainly included Pearson correlation between metabolites, Spearman analysis between samples, and the clustering method for H clusters with complete algorithms. The effect of the gestation period on the metabolite set enrichment and metabolic pathways was analyzed using the SciPy package (version 1.0.0) in PYTHON and Stats package in R with Fisher’s exact test.

A total of 24 pairs of samples with both plasma metabolome and feces microbiome data were investigated to determine the correlation between the shifts in plasma metabolites and the changes in the bacterial communities. These correlations were evaluated via Procrustes analysis and Pearson’s correlation analysis using the SciPy package (version 1.0.0) in PYTHON. The *p*-values were adjusted with FDR, and corrected *p*-values < 0.05 were considered statistically significant.

## Results

### Alpha diversity of fecal bacteria

**FIGURE 1:**
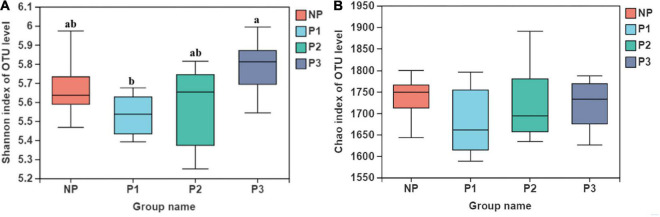
Fecal bacteria alpha diversity indices of donkey feces. **(A)** shannon index; **(B)** Chao index.

Indices of alpha diversity, including the Shannon and Chao indexes, are presented in Figure 1. The bacterial community diversity with the Shannon index was lower in P1 than it was in P3 (*p* < 0.05). However, no difference emerged among different gestation periods for the Chao index (*p* > 0.05).

### Beta diversity of bacteria

**FIGURE 2:**
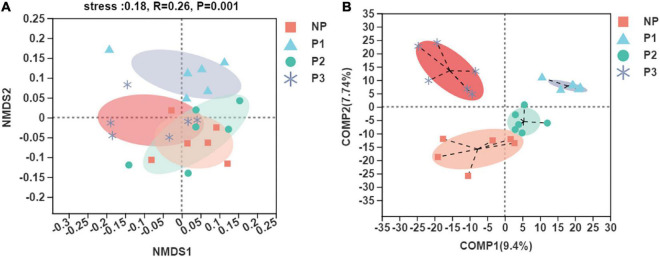
Scatterplot from NMDS **(A)** and PLS-DA **(B)** of OTUs shown the differences in fecal bacteria community structures of donkeys among different gestation periods.

Unweighted UniFrac Non-metric Multidimensional Scaling (NMDS) analysis at the OTU level was conducted to evaluate the extent of the similarity of the fecal bacteria communities (Figure 2A). The results showed that the fecal microbiota was obviously separated between the NP and P1 groups, but it was less segregated between the NP and P2 groups. PLS-DA was then performed to analyze the high-dimensional data to distinguish between the observed values among groups (Figure 2B). The fecal bacteria communities in the NP, P1, P2, and P3 samples clustered separately. An analysis of similarities (ANOSIM) based on UniFrac distances and a non-parametric multivariate analysis of variance (Adonis) were calculated, and the *p*-values (*p* = 0.05 for ANOSIM, *p* = 0.03 for Adonis) further indicated significant differences in the bacterial communities among groups.

### Taxonomic composition of fecal bacteria during gestation

**FIGURE 3:**
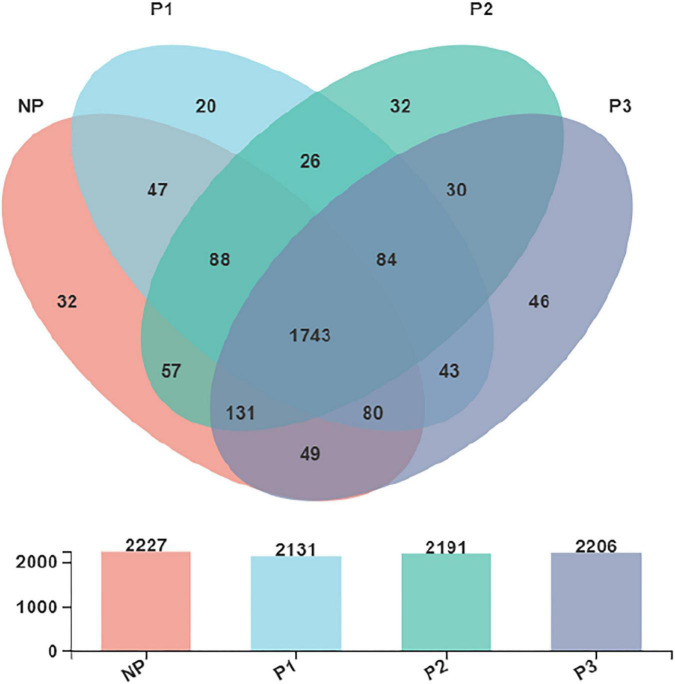
Venn diagram presented the distribution of fecal bacteria community OTUs in donkeys.

The Venn diagram (Figure 3) presented the distribution of bacterial community OTUs. There were 2,227, 2,131, 2,191, and 2,206 OTUs observed in the NP, P1, P2, and P3 groups, respectively. NP shared a bacterial community, including 1,743 OTUs, with P1, P2, and P3.

Bacteria with a relative abundance of ≥1% of the total sequences in at least one of the fecal samples were further analyzed (Table 1). Among the phyla, Firmicutes and Bacteroidetes were detected as the dominant phyla, regardless of the gestational stage, and their total proportions were as high as >80%. Spirochaetota and Fibrobacterota both showed obviously higher relative abundance in both the P1 and P2 groups than in the NP group (*p* < 0.05). In contrast, the relative abundance of Verrucomicrobiota in the P1 and P2 groups was lower than that in the NP group (*p* < 0.05). There were no significant differences among the gestation stages for the relative abundance of other bacteria at the phylum level (*p* > 0.05).

**TABLE 1:** The relative abundance (> 1%) of fecal bacteria community composition in donkeys at the phylum and genus level during gestation period.

Items	NP	P1	P2	P3	*P-value*
**PHYLUM**					
Firmicutes	63.3 ± 4.0	63.8 ± 6.7	66.4 ± 9.6	64.2 ± 6.6	0.92
Bacteroidota	23.4 ± 2.9	24.8 ± 3.8	21.7 ± 6.4	21.3 ± 4.9	0.58
Spirochaetota	3.1 ± 1.1^c^	7.3 ± 3.3^a^	5.1 ± 2.7^b^	5.0 ± 2.2^b^	0.05
Verrucomicrobiota	6.0 ± 5.4^a^	1.4 ± 0.8^c^	3.6 ± 1.4^b^	5.5 ± 5.1^ab^	0.02
Actinobacteriota	1.0 ± 0.3	0.8 ± 0.3	0.8 ± 0.2	1.1 ± 0.2	0.22
Proteobacteria	1.4 ± 2.0	0.2 ± 0.1	0.2 ± 0.1	0.7 ± 0.8	0.28
Patescibacteria	0.7 ± 0.3^a^	0.3 ± 0.1^b^	0.6 ± 0.3^a^	1.0 ± 0.3^a^	0.01
Fibrobacterota	0.3 ± 0.3^b^	1.0 ± 0.6^a^	0.8 ± 0.7^a^	0.2 ± 0.1^b^	0.04
Others	1.8 ± 1.1	1.7 ± 1.0	2.1 ± 1.6	2.2 ± 1.3	0.62
**GENUS**					
unclassified_f_*Lachnospiraceae*	6.27 ± 2.4	7.17 ± 1.5	6.58 ± 1.7	5.50 ± 1.2	0.31
*Lachnospiraceae*_AC2044_group	6.54 ± 1.8	7.41 ± 1.8	5.66 ± 2.2	5.51 ± 2.9	0.46
*Rikenellaceae*_RC9_gut_group	7.98 ± 3.2	4.71 ± 1.6	5.49 ± 1.7	6.16 ± 1.4	0.21
*Streptococcus*	5.38 ± 4.1^b^	4.03 ± 4.3^b^	10.1 ± 6.3^a^	3.68 ± 2.5^b^	0.23
norank_f_*p*-251-o5	3.92 ± 2.9	8.50 ± 4.2	5.41 ± 3.8	4.00 ± 1.8	0.18
*Lachnospiraceae*_UCG-009	4.59 ± 2.5	6.03 ± 2.1	4.86 ± 1.9	5.02 ± 2.5	0.72
*Treponema*	2.91 ± 1.1^c^	7.20 ± 3.3^a^	4.87 ± 2.8^b^	4.81 ± 2.2^b^	0.05
*Ruminococcaceae*_NK4A214_group	4.70 ± 2.1^a^	2.69 ± 0.6^b^	3.69 ± 0.2^ab^	5.08 ± 1.0^a^	0.01
norank_f_norank_o_WCHB1-41	4.71 ± 4.3^a^	1.23 ± 0.7^c^	3.08 ± 1.4^b^	4.43 ± 4.6^a^	0.04
*Christensenellaceae*_R-7_group	3.28 ± 1.1	3.29 ± 0.5	3.09 ± 0.5	3.731 ± 0.6	0.33
Others	49.7 ± 27.3	47.8 ± 25.1	47.2 ± 23.5	52.1 ± 26.8	0.56

NP, no pregnant period; P1, pregnant period 1; P2, pregnant period 2; P3, pregnant period 3. The superscript letters are significantly different at *p* < 0.05.

To further investigate the bacterial community compositions of fecal samples, 10 genera with a mean relative abundance ≥1% of the total sequences in at least one sample were analyzed. The unclassified_f_*Lachnospiraceae* belonging to phylum Bacteroidetes were the most predominant genera in the fecal bacteria. Although the abundance of *Ruminococcaceae*_NK4A214_group was higher in NP than it was in P1 (*p* < 0.05), its abundance increased linearly with the progression of pregnancy (i.e., representing 2.69%, 3.69%, and 5.08% of the bacteria in groups P1, P2, and P3, respectively; *p* < 0.05). The genus *Streptococcus* reached its maximum abundance (peaked) in P2 of pregnancy (10.1%; *p* < 0.05). No significant differences emerged among the gestation stages for the abundance of other genera.

### Bacterial biomarkers of different pregnancy stages

**FIGURE 4:**
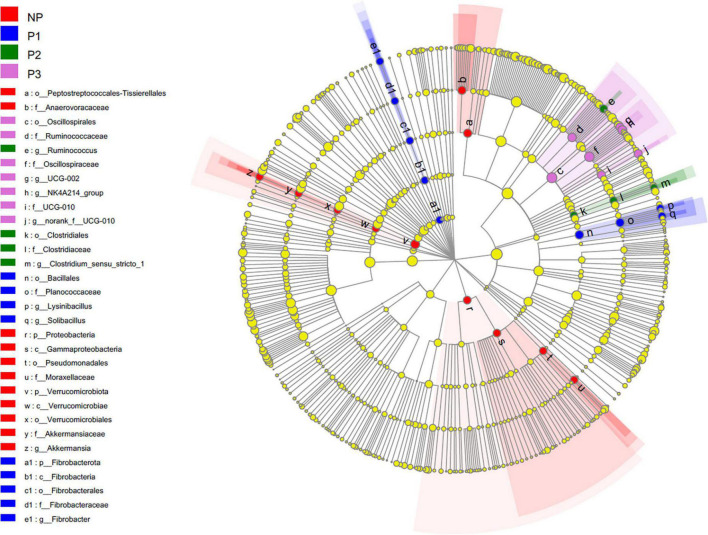
Cladogram plotted from LEfSe analysis indicated the biomarkers of the bacterial community among different gestation periods (*p* < 0.05; LDA score 3.5). NP, no pregnant period; P1, pregnant period 1; P2, pregnant period 2; P3, pregnant period 3.

Linear discriminant analysis effect size (LEfSe) analysis was applied to identify the biomarker species that distinguish the bacterial communities among different pregnancy stages (Figure 4). In the present study, the dominant species from the fecal samples in the NP group were mainly from the classes Verrucomicrobiota, Proteobacteria, Gammaproteobacteria, Akkermansiaceae, Pseudomonadales, Moraxellaceae, Peptostreptococcales-Tissierellales, and Anaerovoracaceae. The biomarker species in P1 belonged to the classes Bacillales, Planococcaceae, Solibacillus, Lysinibacillus, and Fibrobacterota. The dominant species in P2 were predominantly from the classes Clostridiaceae, Clostridium_sensu_stricto_1, and Ruminococcus. The dominant species in P3 were from the classes Oscillospirales, Ruminococcaceae, Ruminococcaceae_NK4A214_group, UCG-002, and UCG-010.

### Potential function capacity of the fecal microbiome during the gestation period

**FIGURE 5:**
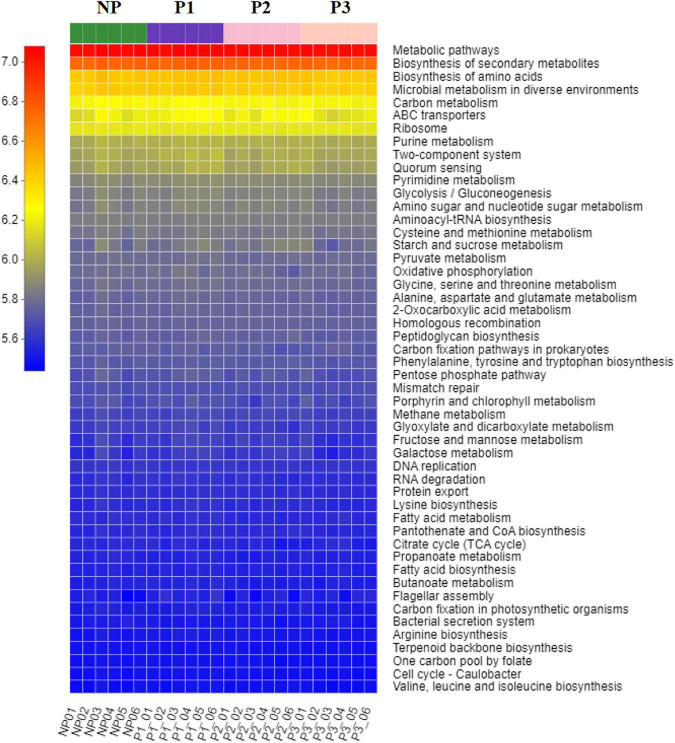
The predicted KEGG pathways within different stages of gestation. NP, no pregnant period; P1, pregnant period 1; P2, pregnant period 2; P3, pregnant period 3. The X-axis represents the sample name; the Y-axis shows the function name of KEGG pathway; the changes of different functional abundances in samples are displayed by color block; the red represents a higher abundance; the blue represents a lower abundance.

PICRUSt software was used to characterize the potential functional capacity of the donkey fecal microbiome with the 16S rRNA data. The relative abundances of KEGG pathways among the NP, P1, P2, and P3 groups were compared in the present study (Figure 5). There was no significant difference for the relative abundance of the top 50 pathways during the gestation period.

### Comparative analysis of donkey plasma metabolites

Plasma samples (*n* = 6 per group) were obtained at different stages of the gestation period with 16S rRNA gene sequencing data. In the present study, 327 quantifiable plasma metabolites were identified for further analysis, including 175 and 152 metabolites from the positive and negative ion modes, respectively. A PCA analysis was performed to evaluate the global changes in the donkey plasma metabolome during different gestation periods (Supplementary Figure 1). There was no significant separation among the metabolic samples in the different groups in the positive ion mode, but such a separation was observed in the positive ion mode.

**FIGURE 6:**
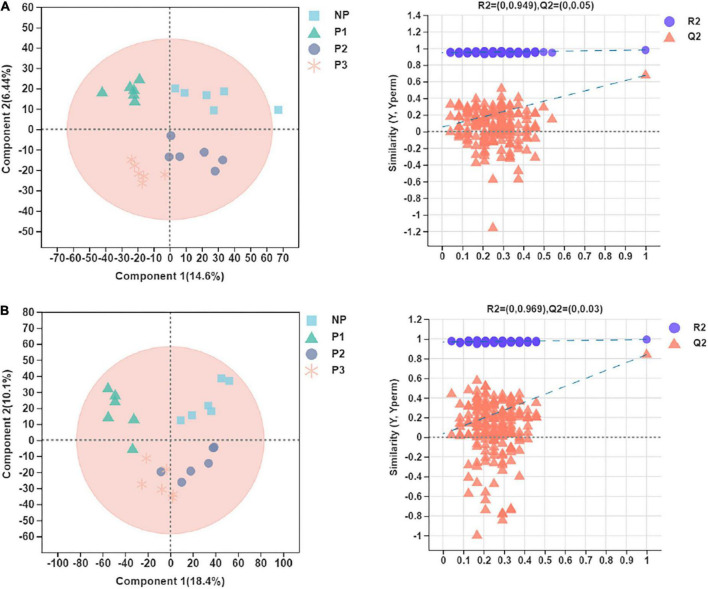
Partial least squares discriminant analysis (PLS-DA) plot and permutation testing of donkey plasma metabolites in comparisons of the different gestation periods following **(A)** positive and **(B)** negative mode ionization.

Score plots of the PLS-DA performed to demonstrate the differentiated metabolites among different pregnant groups and the permutation testing are shown in Figure 6. Almost all the samples in the score plots were within the 95% Hotelling T2 ellipse. Both positive and negative data showed clear separation and discrimination among the different pregnancy groups, indicating that the PLS-DA model can be applied to identify differences. In the positive and negative ion modes of the PLS-DA, 48.8% and 56.5% of the total explained variation in the data set (R^2^X cum) was used to account for 98.1% and 99.3% of the variance in the class separation (R^2^Y cum), and the cross-validated predictive abilities of the model were 67.4% and 84.0% (Q^2^ cum). The permutation test indicated satisfactory effectiveness of the model, with R^2^Y values of 0.949 and 0.969 and Q^2^Y values of 0.05 and 0.03 for the positive and negative mode ionization, respectively.

### Identification and evaluation of differential metabolites

**FIGURE 7:**
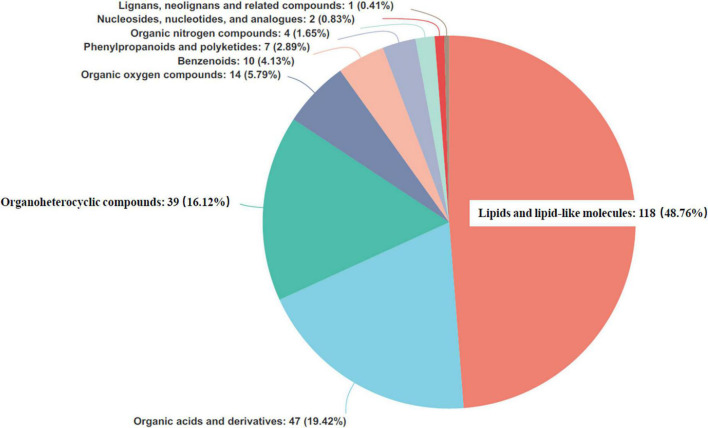
Pie chart based on counts of human urine metabolome database (HMDB) chemical taxonomy for all metabolites identified in the present study (*n* = 24).

Each identified metabolite was searched against synonyms in the HMDB (Figure 7). The compounds covered a range of chemical classes, defined in the HMDB as the chemical “Super Class.” “Lipids and lipid-like molecules,” “organic acids and derivatives,” and “organoheterocyclic compounds” were detected as the dominant metabolites. Their total proportions were found to be as high as >84%.

**FIGURE 8:**
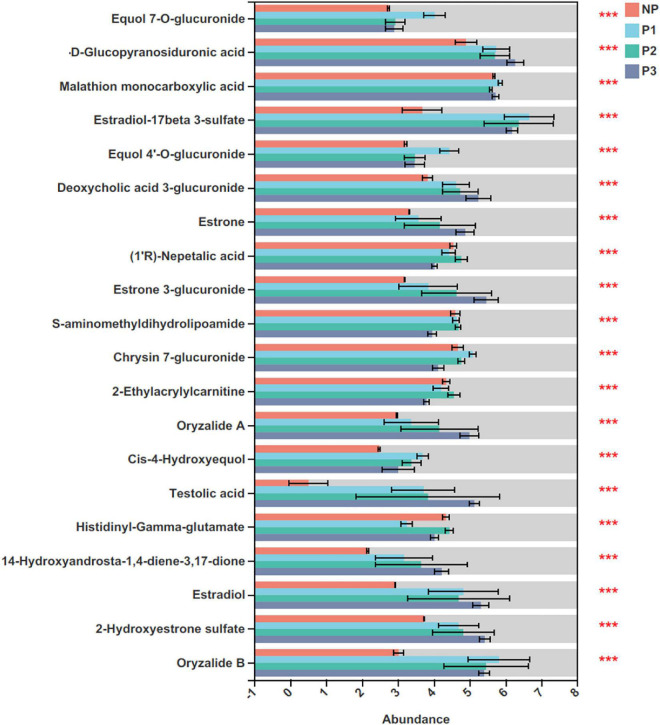
Identification of significant differential metabolites (top 20) in donkey plasma in different gestation periods. NP, no pregnant period; P1, pregnant period 1; P2, pregnant period 2; P3, pregnant period 3; ***p < 0.001.

A total of 76 differential metabolites with FDR-adjusted *p*-values < 0.05 were selected, including 20 and 56 metabolites in the positive and negative ion modes, respectively, from the 24 donkey plasma samples (Supplementary Table 1). The top 20 differential metabolites among distinct gestation periods are presented in Figure 8. As pregnancy progressed, the levels of testolic acid, estradiol-17beta 3-sulfate, equol 7’-o-glucuronide, equol 4’-o-glucuronide, estrone, estrone 3-glucuronide, and estradiol gradually increased.

**FIGURE 9:**
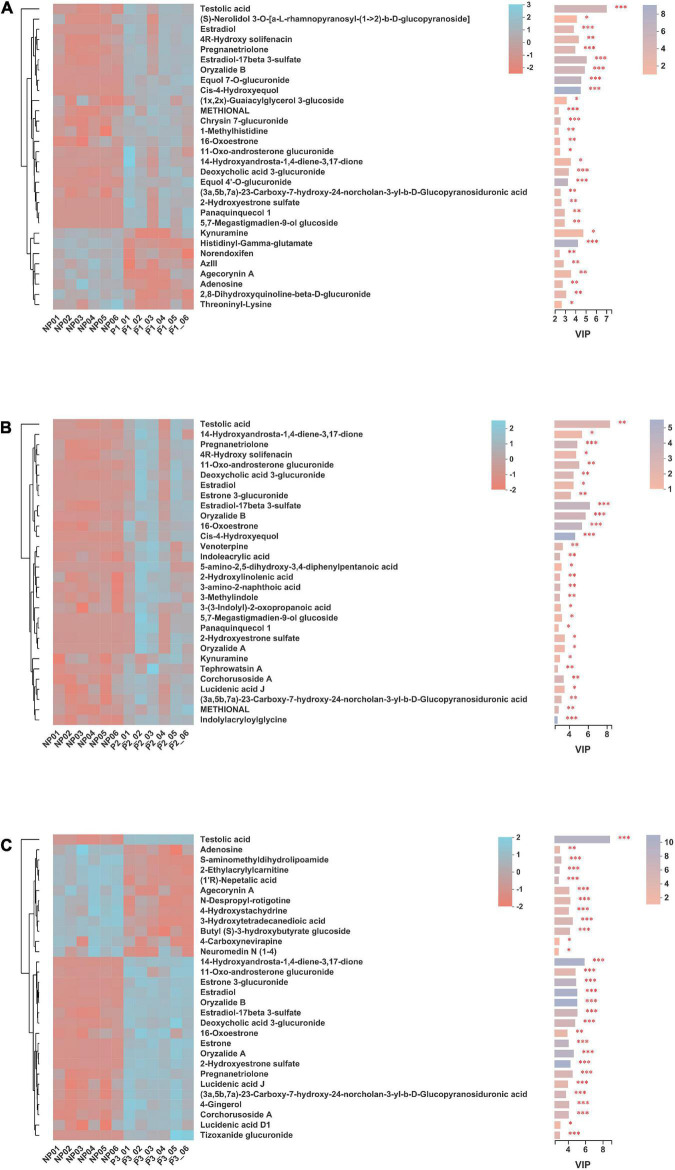
Significantly differential metabolites (top 30) in donkey plasma between **(A)** NP and P1 donkeys, **(B)** NP and P2 donkeys and **(C)** NP and P3 donkeys based on variable importance in the projection (VIP) value under merger mode of positive and negative ion. NP, no pregnant period; P1, pregnant period 1; P2, pregnant period 2; P3, pregnant period 3. The selected metabolites were those with VIP score more than 1. Each row represents a metabolite, each column represents a sample, and the color indicates the relative expression level of the metabolites in the samples. The right side is the metabolite VIP bar graph, the length of bar represents the contribution of the metabolites to the difference between the two groups, the color of the bar means the FDR-adjusted P-value (*p < 0.05; ^**^, *p* < 0.01; ^***^p < 0.001).

Significantly differential metabolites (top 30) in donkey plasma between the NP and P1 groups, NP and P2 groups, and NP and P3 groups are presented in Figures 9A–C, respectively. This study found 103 differentially enriched metabolites between NP and P1; 20 of the metabolites were increased in P1 (FC > 1.2), while 4 were decreased (FC < 0.8). The following metabolites were characterized: carboxylic acids and derivatives (*n* = 16), steroids, and steroid derivatives (*n* = 12), fatty acyls (*n* = 10), prenol lipids (*n* = 7), organonitrogen compounds (*n* = 6), glycerophospholipids (*n* = 3), indoles and derivatives (*n* = 3), flavonoids (*n* = 1), and isoflavonoids (*n* = 1; Supplementary Table 2). The levels of testolic acid, (S)-nerolidol 3-O-[a-L-rhamnopyranosyl-(1- > 2)-b-D-glucopyranoside], 4R-hydroxy solifenacin, oryzalide B, estradiol-17beta 3-sulfate, estradiol, 11-oxo-androsterone glucuronide, pregnanetriolone, cis-4-hydroxyequol, and equol 7-O-glucuronide were increased in the P1 group, with FCs of 7.26, 2.94, 2.32, 1.93, 1.81, 1.65, 1.53, 1.52, 1.49, and 1.47, respectively. The 10 characteristic metabolites with the highest fold changes were steroids and steroid derivatives, prenol lipids, organooxygen compounds, flavonoids, isoflavonoids, carboxylic acids and derivatives, tetrahydroisoquinolines, and fatty acyls (Figure 9A).

**FIGURE 10:**
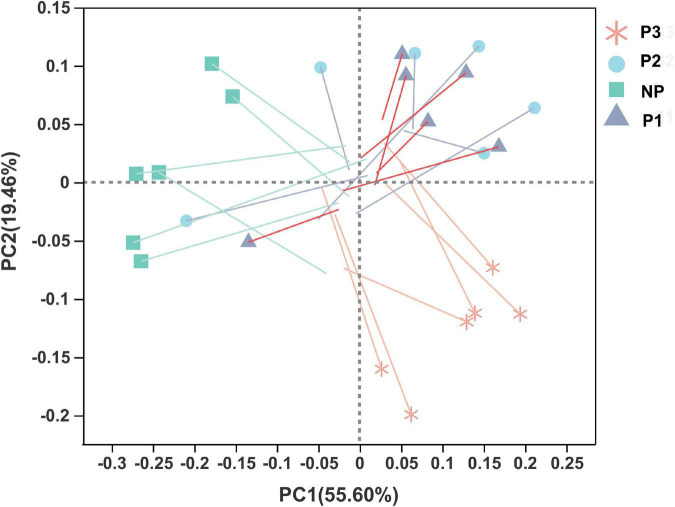
Procrustes analysis to assess the consistency of donkey fecal microbiome and plasma metabolomics profile data. One line and one dot with an arrow represent one sample. Shorter lengths of the lines indicate greater consistency of the four datasets. NP, no pregnant period; P1, pregnant period 1; P2, pregnant period 2; P3, pregnant period 3.

A total of 124 significant variations in metabolites were observed between NP and P2; 64 of them were positively ionized, whereas 60 were negatively ionized (Supplementary Table 3). The varying metabolites involved were fatty acyls (*n* = 20), carboxylic acids and derivatives (*n* = 7), organonitrogen compounds (*n* = 9), indoles and derivatives (*n* = 8), prenol lipids (*n* = 13), steroids and steroid derivatives (*n* = 13), furanoid lignans (*n* = 1), isoflavonoids (*n* = 1), and pyridines and derivatives (*n* = 1). Testolic acid, 4R-hydroxy solifenacin, 11-oxo-androsterone glucuronide, oryzalide B, estradiol-17beta 3-sulfate, 14-hydroxyandrosta-1,4-diene-3,17-dione, estradiol, pregnanetriolone, estrone 3-glucuronide, and venoterpine were the top 10 variations in metabolites in the P2 group, with FCs of 7.53, 2.09, 1.90, 1.81, 1.73, 1.69, 1.61, 1.49, 1.46, and 1.40, respectively. The characteristic metabolites with the higher change ratios were tetrahydroisoquinolines, glycerolipids, prenol lipids, steroids, and steroid derivatives (Figure 10B).

As shown in Supplementary Table 4, 135 differentially metabolites between the NP and P3 groups were identified using a VIP threshold of 1 (*P* < 0.05). 26 metabolites were significantly increased and 8 were significantly decreased. The changing metabolites included steroids and steroid derivatives (*n* = 17), prenol lipids (*n* = 7), indoles and derivatives (*n* = 5), glycerophospholipids (*n* = 13), fatty acyls (*n* = 17), carboxylic acids and derivatives (*n* = 15), and isoflavonoids (*n* = 1; Supplementary Table 2). The levels of testolic acid, deoxycholic acid 3-glucuronide, 14-hydroxyandrosta-1,4-diene-3,17-dione, estradiol, oryzalide B, estrone 3-glucuronide, oryzalide A, estradiol-17beta 3-sulfate, pregnanetriolone, and 16-oxoestrone were increased in the P3 group, with FCs of 10.06, 2.08, 1.95, 1.82, 1.79, 1.72, 1.68, 1.68, 1.56, and 1.55, respectively. The top 10 characteristic metabolites with a higher shift ratio were steroids and steroid derivatives and prenol lipids.

### Metabolic pathway enrichment analysis

**FIGURE 11:**
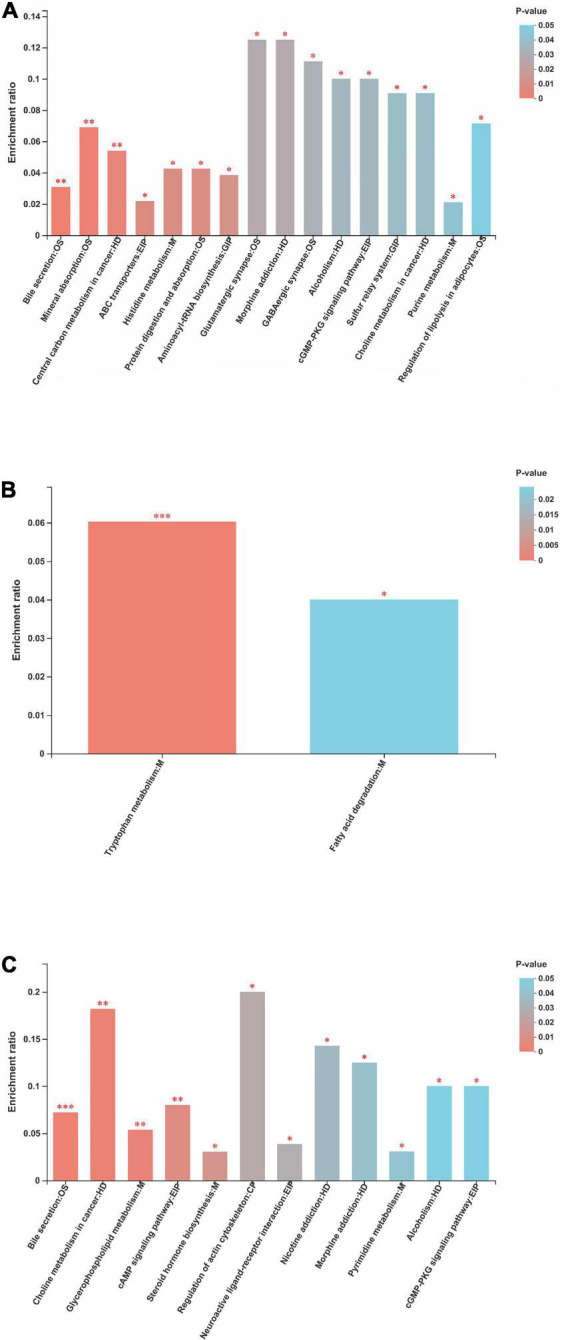
Metabolic pathway enrichment of differential metabolites between **(A)** NP and P1 group, between **(B)** NP and P2 group, between **(C)** NP and P3 group. NP, no pregnant period; P1, pregnant period 1; P2, pregnant period 2; P3, pregnant period 3; OS, M, HD, GIP, EIP and CP are the class names of the metabolic pathways in the KEGG annotation. OS, Organismal Systems; M, Metabolism; HD, Human Diseases; GIP, Genetic Information Processing; EIP, Environmental Information Processing; CP, Cellular Processes. *p < 0.05; ^**^p < 0.01; ^***^p < 0.001.

The metabolites identified were assigned to the KEGG second-grade pathways. For the “metabolism” term, the top priority was “lipid metabolism,” followed by “amino acid metabolism,” “digestive system,” “nervous system,” “metabolism of cofactors and vitamins,” and “membrane transport” (Supplementary Figure 2). The metabolic KEGG pathways with *p* < 0.05 are considered the most relevant pathways involved in the present study (Figure 11). The enrichment analysis showed that such metabolic pathways as bile secretion, ABC transporters, histidine metabolism, purine metabolism, protein digestion and absorption, mineral absorption, aminoacyl-tRNA biosynthesis, and glutamatergic synapse were significantly changed between the NP and P1 groups. Only two metabolic pathways (tryptophan metabolism and fatty acid degradation) were altered between the NP and P2 groups. The metabolic pathways, including bile secretion, glycerophospholipid metabolism, steroid hormone biosynthesis, pyrimidine metabolism, regulation of actin cytoskeleton, and cGMP-PKG signaling pathway, were changed between the NP and P3 groups.

### Correlation between fecal bacteria and plasma metabolomics

A Procrustes analysis was conducted to evaluate the consistency of the data from donkey fecal bacteria and plasma metabolomics profiling; the results showed that the similarity among the four datasets was low (*p* = 0.40, Figure 10).

**FIGURE 12:**
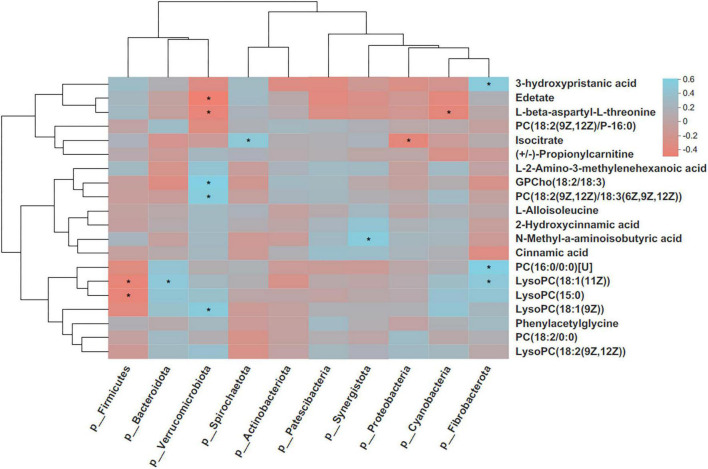
Correlation heatmap of differentially donkey fecal phylum and enriched metabolites. Each row in the graph represents a metabolite, each column represents a microbiota phylum, the color in the graph indicates the Pearson coefficient between the 10 bacterial phylum and the 20 metabolite, and the pale blue indicates positive correlation. The brick red is representative negative correlation. The darker color indicate the 16 greater the correlation. *Indicates a significant correlation at *p* < 0.05. PC, phosphatidylcholine; LysoPC, Lysophosphatidylcholine.

The possible correlations between changed plasma metabolites and fecal bacterial phyla were determined based on Spearman’s correlation (Figure 12). The fecal Firmicutes was negatively correlated with lysophosphatidylcholine (LysoPC)[18:1(11Z)] and LysoPC(15:0)s (*p* < 0.05), whereas Bacteroidota was positively correlated with LysoPC[18:1(11Z)] (*p* < 0.05). Fecal Verrucomicrobiota was positively correlated with L-proline, L-glutamine, GPCho(18:2/18:3), phosphatidylcholine (PC)[18:2(9Z,12Z)/18:3(6Z,9Z,12Z)], and LysoPC[18:1(9Z)] (*p* < 0.05), whereas it was negatively correlated with L-beta-aspartyl-L-threonine (*p* < 0.05). There were significant correlations between Spirochaetota and isocitrate and between Synergistota and N-Methyl-a-aminoisobutyric acid (*P* < 0.05). In addition, Fibrobacterota was positively correlated with 3-hydroxypristanic acid, PC(16:0/0:0)[U], and LysoPC[18:1(11Z)] (*p* < 0.05).

## Discussion

The gestation length for donkeys is long, and it has been reported as 333–395 days (Miragaya et al., 2018); the gestation period is an important part of donkey reproduction stewardship. The pregnancy period is accompanied by gut microbiota remodeling that participates in modulating maternal physiology and metabolism (Koren et al., 2012). These modulations could be important in maternal health and fetal nourishment; however, little in-depth information from donkeys is available to understand the role of gut microbiota and related physiological metabolites during this critical time. In the present study, the remodeling of fecal microbiota and corresponding changes in plasma metabolites over the course of pregnancy were presented.

In the present results, it was found that the diversity of the bacterial community (Shannon index) increased throughout the gestation period, indicating a dramatic shift in fecal bacteria between pregnant and non-pregnant donkeys. The gut microbiota diversity is well known to improve the stability and performance of communities, and it has been proposed as an important biomarker of health and metabolic capacity (Clarke et al., 2014; Tap et al., 2015; Li et al., 2018). Therefore, diverse bacteria probably provide many metabolic capacities and functional redundancy in pregnant donkeys, ensuring the sufficient supply of nutrients for maternal health and fetal development. The beta-diversity results with NMDS and the PLS-DA analysis demonstrated significant differences in the bacterial composition among the stages of gestation, especially the early stage. The results were consistent with those of previous studies that reported obvious changes in microbiota composition during pregnancy in donkeys (Li et al., 2022). However, it is well known that gut microbiota can be affected by various host and environmental factors (Grzeskowiak et al., 2012). Host genetics, diet, and individual differences are difficult to control to understand the specific changes in the gut microbiota (Wu et al., 2011; Goodrich et al., 2014; Ji et al., 2019). The samples obtained in the present study were collected from different animals, and the variability derived from individual differences may not have been eliminated. As a result, less segregation in the NP and P2 groups was found in the current NMDS analysis. Further research with the same individuals is needed to assess changes in gut microbiota in the same individuals.

Gut bacterial communities generally play a significant role in the functioning and health of their hosts (Zhang et al., 2020). In the present study, Firmicutes and Bacteroidetes were prominent in the donkey fecal microbiota in all samples. This agrees with the results reported by Liu et al. (2020) to the effect that the two dominant bacteria in the domestic donkey hindgut came from the phyla Bacteroidetes and Firmicutes. Firmicutes is important for carbohydrate breakdown in the donkey gut, and this is correlated with the efficient hydrolysis of plant biomass and feed utilization (Myer et al., 2015). Bacteroides are the primary degraders of carbohydrate-based polysaccharides (Sonnenburg et al., 2010). They contribute to the release of energy from dietary fiber and starch, and they are likely to be a major source of propionate (Liu G. Q. et al., 2019). In the present study, both Firmicutes and Bacteroidetes remained remarkably stable throughout the gestation period, which is in line with the previous studies by Koren et al. (2012) and Jost et al. (2014), who observed no detectable shifts in the abundance of Firmicutes and Bacteroidetes during pregnancy in humans. The variation in the relative abundance of specific bacterial taxa is usually associated with dietary constituents (Carrothers et al., 2015). Thus, when the donkeys were fed a consistent diet in the current study, no alteration of Firmicutes and Bacteroidetes was observed.

Several other studies have reported pregnancy-induced alterations in gut bacterial composition (Dethlefsen and Relman, 2011; Khan et al., 2016; Li et al., 2022). The present results also demonstrated that the relative abundance of Spirochaetota and Fibrobacterota were promoted by pregnancy. The increased abundance of phyla Spirochaetota in pregnant donkeys might be mainly attributed to the enrichment of the genus *Treponema*. Until now, some *Treponema* species have been identified from the gastrointestinal tracts of mammal animals, including pigs and cows (Stanton and Canale-Parola, 1980; Ji et al., 2019). *Treponema* has been reported to be capable of digesting cellulose (Bekele et al., 2011). In addition, the phylum Fibrobacterota is recognized as a major bacterial degrader of lignocellulosic material in the herbivore gut (Ransom-Jones et al., 2012). In contrast, the present results revealed a decline in the abundance of Verrucomicrobiota and Proteobacteria as gestation progressed. Consistent with the decline in the phylum Verrucomicrobiota, the relative abundance of genera norank_f_norank_o_WCHB1-41 also decreased during pregnancy. In addition, the bacterial phyla Verrucomicrobiota and Proteobacteria belong to the members of aerobic methanotrophic capabilities (Hernandez et al., 2021). The reduction in Verrucomicrobiota and Proteobacteria may indicate less methane production in the donkey hindgut, but further study is needed to investigate this inference.

The LEfSe analysis performed in the present study showed that the classes related to plant cell wall breakdown increasing in the pregnancy period (P1, P2, and P3) included Fibrobacterota, Ruminococcus, Ruminococcaceae, *Ruminococcaceae*_ NK4A214_group, *Ruminococcaceae*_UCG-002, and *Ruminococcaceae*_UCG-010. This implies that dietary fiber compounds can be metabolized efficiently by the gut bacteria during the gestation period for the potential benefit of the donkeys and their fetuses. The present result is consistent with the previous study reported by Ji et al. (2019) in sows, which showed numerous changes during the pregnancy of sows, where most of the bacteria that increased in abundance with the progression of pregnancy could digest polysaccharides and lignocellulose materials.

In concordance with the lack of significant shifts in the predominant phyla Firmicutes and Bacteroidetes, the potential functional capacity of the gut microbiome predicted by PICRUSt also showed no obvious change among different gestation periods in donkeys. Bacteria related to metabolic pathways, biosynthesis of secondary metabolites, biosynthesis of amino acids, microbial metabolism in diverse environments, and carbon metabolism were the top five metabolic functions based on the results from the 16S rRNA gene sequencing at KEGG taxonomy. This implies that the functional capacity of donkey gut microbiota appeared to be relatively stable over different stages of gestation, probably providing an adequate supply of nutrients available for fetal growth and maternal health.

The physiological biochemical metabolites of the blood are significant biomarkers for monitoring donkey health and physiological responses (Liao et al., 2021; Li et al., 2022). To the best of our knowledge, the current study is the first integrative research report that has used the LC-MS-based metabolomics approach to investigate the plasma metabolic variations in donkeys throughout the pregnancy period. Compared with the NP, during the gestation period, the metabolites were enriched in pathways matched to bile secretion, mineral absorption, amino acid metabolism, protein digestion and absorption, purine metabolism, fatty acid degradation, glycerophospholipid metabolism, steroid hormone biosynthesis, and pyrimidine metabolism.

As the key regulator of metabolism, the important role of bile acids in gestational changes in metabolism is becoming apparent (Mcilvride et al., 2017). In the present study, the enrichment of bile secretion was detected at the early and late stages of pregnancy, suggesting a progressive rise in plasma bile acid synthesis with advancing gestation. The pathway for bile acid synthesis usually results in the production of primary bile acids, cholic acids, and deoxycholic acids (Mcilvride et al., 2017). Previous studies have reported that the conjugated bile acids are generally higher in the serum of pregnant women (Fulton et al., 1983; Brites, 2002; Castano et al., 2006). In agreement with the result, the metabolites of deoxycholic acid 3-glucuronide in donkey plasma were significantly increased in the pregnant groups. The present results provide evidence that bile secretion plays an important role in mediating host metabolism during the perinatal period.

The changed metabolites in the current study were also enriched in pathways matched to ABC transporters, amino acid metabolism (histidine and tryptophan), purine metabolism, protein digestion and absorption, mineral absorption, aminoacyl-tRNA biosynthesis, fatty acid degradation, glycerophospholipid metabolism, and pyrimidine metabolism. As high energy consumption is associated with fetal growth and development, these changes are advantageous to energy delivery and likely to support fetal growth during pregnancy in donkeys (Luan et al., 2014). Furthermore, the physiological changes that occur during pregnancy are the result of steroid hormone biosynthesis, which is necessary to support the developing fetus (Napso et al., 2018).

In the current study, the KEGG pathways of steroid hormone biosynthesis were also enriched, resulting in progressive rises in estradiol, estradiol-17-beta-3-sulfate, estrone, estrone-3-glucuronide and pregnanetriolone among pregnant donkeys. Pregnancy maintenance depends primarily on the interactions of neuronal and hormonal factors (Feldt-Rasmussen and Mathiesen, 2011). Maternal hormone alterations are critical in modulating the immunologic and metabolic changes required for a healthy pregnancy outcome (Tal and Taylor, 2021). Furthermore, the fetus and placenta both produce steroids into the maternal circulation, as well as stimulating the generation of maternal hormones (Tal and Taylor, 2021). It has been found that, as pregnancy progresses, the placenta produces more estradiol-17-beta from the conversion of circulating maternal and fetal dihydroepiandrostenediene sulfate (Siiteri and MacDonald, 1966). However, this is inconsistent with our finding that the metabolites of estradiol and estradiol-17-beta-3-sulfate in donkey plasma were higher in the P1, P2, and P3 groups than they in the NP group. Estradiol concentrations increased in a pattern indicating a definitive increase in intrauterine steroidogenesis. In general, estrone originates primarily from maternal sources, including the ovaries and adrenals (Lindberg et al., 1974). The placenta then produces increasing quantities of estrone from the conversion of circulating maternal and fetal dihydroepiandrostenediene sulfate (Tulchinsky and Hobel, 1973). In a previous study by Tulchinsky et al. (1972), the estrone usually remained within the luteal phase range at the early stage of gestation. Concentrations of estrone increase gradually as pregnancy progresses; therefore, in the present study, there was only a significant difference for the estrone metabolite between the NP and P3 groups. The presence of pregnanetriolone is characteristic of adrenal hyperplasia (Lahoud et al., 1976); however, the specific effect of pregnanetriolone increasing within the gestation period is unknown. This topic requires further investigation.

In this study, the metabolite–microbial relationships were assessed by correlation analysis, and a co-relationship between the plasma metabolites and fecal microbiota was presented. LysoPC is mainly derived from the oxidation of very-low-density lipoprotein and the hydrolysis of lecithin (Ismaeel and Qadri, 2021). LysoPCs are bioactive proinflammatory lipids that have been linked to organismal oxidative stress and inflammation (Sevastou et al., 2013; Liang et al., 2020). Phospholipids, such as PC, are major components of cellular membranes and play a key role in signal transduction; they also make up the majority of lipoproteins (Zeisel, 1993; Rauschert et al., 2019). In the present study, the donkey plasma metabolites of LysoPC[18:1(11Z)], LysoPC[18:1(9Z)], PC[18:2(9Z,12Z)/18:3(6Z,9Z,12Z)], and PC(16:0/0:0)[U] were positively correlated with the bacteria of Bacteroidota, Verrucomicrobiota, and Fibrobacterota. These results showed that both LysoPCs and PC play a key role in mediating host–gut microbiome interactions and shaping gut bacteria and host metabolism during the gestation period. Moreover, the present findings require prospective investigation in mechanistic studies before more definite conclusions on the relationship between LysoPC, PC, and gut bacteria in pregnancy can be drawn.

In summary, the present study suggested that the progression of pregnancy is associated with changes in the composition of donkey gut bacteria and the bacteria’s functional capacities. The bacteria involved in the degradation of carbohydrates, including bacteria in the phyla Spirochaetota and Fibrobacterota, were promoted by pregnancy. The host plasma metabolome was also significantly altered. Furthermore, we found a significant correlation between the changes in the donkey fecal microbiome and alterations in host plasma metabolite features. These results indicated that host–bacterial interactions during the gestation period influence host metabolism, leading to beneficial host alterations to provide a sufficient supply of nutrients and energy for fetal growth and maternal health. However, as a limitation of the study, the samples obtained from different animals may result in variability derived from individual differences. In addition, the causality and the underlying mechanisms of interaction between the changes in the gut microbiome and the shifts in host plasma metabolites have not been elucidated. Further investigation is needed to respond to these remaining questions.

## Data availability statement

The datasets presented in this study can be found in online repositories. The names of the repository/repositories and accession number(s) can be found below: https://www.ncbi.nlm.nih.gov/, PRJNA830593.

## Ethics statement

The animal study was reviewed and approved by the Animal Welfare Committee of Liaocheng University. Written informed consent was obtained from the owners for the participation of their animals in this study.

## Author contributions

ZZ designed research and drafted the manuscript. ZZ, BH, YW, and YZ performed the experiments. MZ and CW reviewed the manuscript. All authors read and approved the submitted manuscript.

## Footnotes

1
https://cloud.majorbio.com

## References

[B1] Bekele A. Z. Satoshi K. Yasuo K. (2011). Phylogenetic diversity and dietary association of rumen treponema revealed using group-specific 16s rrna gene-based analysis. *FEMS Microbiol. Lett.* 316 51–60. 21204927 10.1111/j.1574-6968.2010.02191.x

[B2] Blachier F. Beaumont M. Andriamihaja M. Davila A. M. Lan A. Grauso M. (2017). Changes in the luminal environment of the colonic epithelial cells and physiopathological consequences. *Am. J. Pathol.* 187 476–486. 28082121 10.1016/j.ajpath.2016.11.015

[B3] Brites D. (2002). Intrahepatic cholestasis of pregnancy: changes in maternal-fetal bile acid balance and improvement by ursodeoxycholic acid. *Ann. Hepatol.* 1 20–28. 15114292

[B4] Carrothers J. M. York M. A. Brooker S. L. Lackey K. A. Williams J. E. Shafii B. (2015). Fecalmicrobial community structure is stable over time and related to variation in macronutrient and micronutrient intakes in lactating women. *J. Nutr.* 145 2379–2388. 26311809 10.3945/jn.115.211110 PMC4580954

[B5] Castano G. Lucangioli S. Sookoian S. Mesquida M. Lemberg A. Di Scala M. (2006). Bile acid profiles by capillary electrophoresis in intrahepatic cholestasis of pregnancy. *Clin. Sci. (Lond.)* 110 459–465. 16356162 10.1042/CS20050302

[B6] Cheng C. Wei H. Yu H. Xu C. Jiang S. Jian P. (2018). Metabolic syndrome during perinatal period in sows and the link with gut microbiota and metabolites. *Front. Microbiol.* 9:1989. 10.3389/fmicb.2018.01989 30197635 PMC6117386

[B7] Clarke S. F. Murphy E. F. O’Sullivan O. Lucey A. J. Humphreys M. Hogan A. (2014). Exercise and associated dietary extremes impact on gut microbial diversity. *Gut* 63 1913–1920. 25021423 10.1136/gutjnl-2013-306541

[B8] Dethlefsen L. Relman D. A. (2011). Incomplete recovery and individualized responses of the human distal gut microbiota to repeated antibiotic perturbation. *Proc. Natl. Acad. Sci. U.S.A.* 108 4554–4561. 20847294 10.1073/pnas.1000087107 PMC3063582

[B9] DiGiulio D. B. Callahan B. J. McMurdie P. J. Costello E. K. Lyell D. J. Robaczewska A. (2015). Temporal and spatial variation of the human microbiota during pregnancy. *Proc. Natl. Acad. Sci. U.S.A.* 112 11060–11065. 10.1073/pnas.1502875112 26283357 PMC4568272

[B10] Feldt-Rasmussen U. Mathiesen E. R. (2011). Endocrine disorders in pregnancy: physiological and hormonal aspects of pregnancy. *Best Pract. Res. Clin. Endocrinol. Metab.* 25 875–884. 22115163 10.1016/j.beem.2011.07.004

[B11] Franks I. (2012). The gut microbiota is profoundly altered over the course of pregnancy. *Nat. Rev. Gastroenterol. Hepatol.* 9:560. 10.1038/nrgastro.2012.163 22907163

[B12] Fulton I. C. Douglas J. G. Hutchon D. J. Beckett G. J. (1983). Is normal pregnancy cholestatic? *Clin. Chim. Acta* 130 171–176. 6872255 10.1016/0009-8981(83)90114-6

[B13] Goodrich J. K. Waters J. L. Poole A. C. Sutter J. L. Koren O. Blekhman R. (2014). Human genetics shape the gut microbiome. *Cell* 159 789–799. 25417156 10.1016/j.cell.2014.09.053 PMC4255478

[B14] Grzeskowiak L. Collado M. C. Mangani C. Maleta K. Laitinen K. Ashorn P. (2012). Distinct gut microbiota in Southeastern African and Northern European infants. *J. Pediatr. Gastroenterol. Nutr.* 54 812–816. 10.1097/MPG.0b013e318249039c 22228076

[B15] Hernandez M. Vaksmaa A. Horn M. A. Niemann H. Cruz S. G. (2021). Methanotrophs: discoveries, environmental relevance, and a perspective on current and future applications. *Front. Microbiol.* 12:678057. 10.3389/fmicb.2021.678057 34054786 PMC8163242

[B16] Huang X. Gao J. Zhao Y. He M. Ke S. Wu J. (2019). Dramatic remodeling of the gut microbiome around parturition and its relationship with host serum metabolic changes in sows. *Front. Microbiol.* 10:2123. 10.3389/fmicb.2019.02123 31572329 PMC6751307

[B17] Ismaeel S. Qadri A. (2021). Atp release drives inflammation with lysophosphatidylcholine. *ImmunoHoriz* 5 219–233. 10.4049/immunohorizons.2100023 33911018

[B18] Ji Y. J. Li H. Xie P. F. Li Z. H. Li H. W. Yin Y. L. (2019). Stages of pregnancy and weaning influence the gut microbiota diversity and function in sows. *J. Appl. Microbiol.* 127 867–879. 10.1111/jam.14344 31237071 PMC6852164

[B19] Jost T. Lacroix C. Braegger C. Chassard C. (2014). Stability of the maternal gut microbiota during late pregnancy and early lactation. *Curr. Microbiol.* 68 419–427. 10.1007/s00284-013-0491-6 24258611

[B20] Khan I. Azhar E. I. Abbas A. T. Kumosani T. Barbour E. K. Raoult D. (2016). Metagenomic analysis of antibiotic-induced changes in gut microbiota in a pregnant rat model. *Front. Pharmacol.* 7:104. 10.3389/fphar.2016.00104 27199748 PMC4849429

[B21] Koren O. Goodrich J. K. Cullender T. C. Spor A. Laitinen K. Bäckhed H. K. (2012). Host remodeling of the gut microbiome and metabolic changes during pregnancy. *Cell* 150 470–480. 22863002 10.1016/j.cell.2012.07.008 PMC3505857

[B22] Lahoud H. J. Luttrell B. M. Steinbeck A. W. (1976). Pregnanetriolone, a normal steroid metabolite: its excretion by normal, Cushing’s syndrome and congenital adrenal hyperplasia subjects. *Steroids* 27 211–223. 10.1016/0039-128x(76)90098-2 1273887

[B23] Li H. Qu J. Li T. Wirth S. Zhang Y. Zhao X. (2018). Diet simplification selects for high gut microbial diversity and strong fermenting ability in high-altitude pikas. *Appl. Microbiol. Biotechnol.* 102 6739–6751. 10.1007/s00253-018-9097-z 29862448

[B24] Li Y. Ma Q. S. Liu G. Q. Zhang Z. W. Zhan Y. D. Zhu M. X. (2022). Metabolic alternations during gestation in Dezhou donkeys and the link to the gut microbiota. *Front. Microbiol.* 13:801976. 10.3389/fmicb.2022.801976 35369472 PMC8969422

[B25] Liang L. Rasmussen M. Piening B. Shen X. Chen S. Rst H. (2020). Metabolic dynamics and prediction of gestational age and time to delivery in pregnant women. *Cell* 181 1680–1692.e15. 32589958 10.1016/j.cell.2020.05.002 PMC7327522

[B26] Liao Q. Li Z. Han Y. Deng L. (2021). Comparative analysis of serum mineral and biochemical parameter profiles between late pregnant and early lactating jennies. *J. Equine Vet. Sci.* 99:103401. 10.1016/j.jevs.2021.103401 33781411

[B27] Lindberg B. S. Johansson E. D. Nilsson B. A. (1974). Plasma levels of nonconjugated oestrone, oestradiol-17beta and oestriolduring uncomplicated pregnancy. *Acta Obstet. Gyn. Scan.* 32 21–36. 10.3109/00016347409156390 4527049

[B28] Liu C. Wu H. Liu S. J. Chai S. T. Zhou Z. M. (2019). Dynamic alterations in yak rumen bacteria community and metabolome characteristics in response to feed type. *Front. Microbiol.* 10:1116. 10.3389/fmicb.2019.01116 31191470 PMC6538947

[B29] Liu G. Q. Bou G. Su S. F. Xing J. Y. Qu H. L. Zhang X. Z. (2019). Microbial diversity within the digestive tract contents of Dezhou donkeys. *PLoS One* 14:e0226186. 10.1371/journal.pone.0226186 31834903 PMC6910686

[B30] Liu H. B. Hou C. L. Li N. Zhang X. Y. Zhang G. L. Yang F. Y. (2019). Microbial and metabolic alterations in gut microbiota of sows during pregnancy and lactation. *FASEB J.* 33 4490–4501. 30653349 10.1096/fj.201801221RR

[B31] Liu H. Zhao X. Han X. Xu S. Zhao L. Hu L. (2020). Comparative study of gut microbiota in tibetan wild asses (equus kiang) and domestic donkeys (equus asinus) on the qinghai-tibet plateau. *PeerJ* 8:e9032. 10.7717/peerj.9032 32547852 PMC7276150

[B32] Luan H. Meng N. Liu P. Feng Q. Lin S. Fu J. (2014). Pregnancy-induced metabolic phenotype variations in maternal plasma. *J. Proteome Res.* 13 1527–1536. 24450375 10.1021/pr401068k

[B33] Martin-Rosset W. (2018). Donkey nutrition and feeding: nutrient requirements and recommended allowancesda review and prospect. *J. Equine Vet. Sci.* 65 75–85.

[B34] Mcilvride S. Dixon P. H. Williamson C. (2017). Bile acids and gestation. *Mol. Aspects Med.* 56 90–100. 28506676 10.1016/j.mam.2017.05.003

[B35] Miragaya M. H. Neild D. M. Alonso A. E. (2018). A review of reproductive biology and biotechnologies in donkeys. *J. Equine Vet. Sci.* 65 55–61. 10.1016/j.jevs.2017.12.005

[B36] Motosko C. C. Bieber A. K. Pomeranz M. K. Stein J. A. Martires K. J. (2017). Physiologic changes of pregnancy: a review of the literature. *Int. J. Dermatol.* 3 219–224. 10.1016/j.ijwd.2017.09.003 PMC5715231 29234716

[B37] Myer P. R. Smith T. Wells J. E. Kuehn L. A. Freetly H. C. Forster R. J. (2015). Rumen microbiome from steers differing in feed efficiency. *PLoS One* 10:e0129174. 10.1371/journal.pone.0129174 26030887 PMC4451142

[B38] Napso T. Yong H. E. López-Tello J. Sferruzzi-Perri A. N. (2018). The role of placental hormones in mediating maternal adaptations to support pregnancy and lactation. *Front. Physiol.* 9:1091. 10.3389/fphys.2018.01091 30174608 PMC6108594

[B39] National Research Council Donkey and other equids (2007). *Nutrient Requirements of Horses; Horses, N.R.C. (U. S.) C. on 351 N.R. of, Ed.* Washington, DC: National Academies Press, 268–279.

[B40] Nelson D. B. Rockwell L. C. Prioleau M. D. Goetzl L. (2016). The role of the bacterial microbiota on reproductive and pregnancy health. *Anaerobe* 42 67–73. 27612939 10.1016/j.anaerobe.2016.09.001

[B41] Ransom-Jones E. Jones D. L. Mccarthy A. J. Mcdonald J. E. (2012). The fibrobacteres: an important phylum of cellulose-degrading bacteria. *Microb. Ecol.* 63 267–281. 10.1007/s00248-011-9998-1 22213055

[B42] Rauschert S. Gázquez A. Uhl O. Kirchberg F. F. Demmelmair H. Ruíz-Palacios M. (2019). Phospholipids in lipoproteins: compositional differences across VLDL, LDL, and HDL in pregnant women. *Lipids Health Dis.* 18:20. 10.1186/s12944-019-0957-z 30670033 PMC6343318

[B43] Santos A. S. Rodrigues M. A. M. Bessa R. J. B. Ferreira L. M. Martin-Rosset W. (2011). Understanding the equine cecum-colon ecosystem: current knowledge and future perspectives. *Animal* 5 48–56. 10.1017/S1751731110001588 22440701

[B44] Sevastou I. Kaffe E. Mouratis M. A. Aidinis V. (2013). Lysoglycerophospholipids in chronic inflammatory disorders: the PLA(2)/LPC and ATX/LPA axes. *Biochim. Biophys. Acta.* 1831 42–60. 10.1016/j.bbalip.2012.07.019 22867755

[B45] Siiteri P. K. MacDonald P. C. (1966). Placental estrogen biosynthesis during human pregnancy. *J. Clin. Endocr. Metab.* 26 751–761. 4223909 10.1210/jcem-26-7-751

[B46] Sonnenburg E. D. Zheng H. Joglekar P. Higginbottom S. K. Firbank S. J. Bolam D. N. (2010). Specificity of polysaccharide use in intestinal *Bacteroides* species determines diet-induced microbiota alterations. *Cell* 141 1241–1252. 10.1016/j.cell.2010.05.005 20603004 PMC2900928

[B47] Stanton T. B. Canale-Parola E. (1980). *Treponema bryantii* sp. nov. a rumen spirochete that interacts with cellulolytic bacteria. *Arch. Microbiol.* 127 145–156. 10.1007/BF00428018 7425785

[B48] Tal R. Taylor H. S. (2021). “Endocrinology of pregnancy,” in *Endotext [Internet]*, eds Feingold K. R. Anawalt B. Boyce A. (South Dartmouth, MA: MDText.com, Inc).

[B49] Tap J. Furet J. P. Bensaada M. Philippe C. Roth H. Rabot S. (2015). Gut microbiota richness promotes its stability upon increased dietary fibre intake in healthy adults. *Environ. Microbiol.* 17 4954–4964. 10.1111/1462-2920.13006 26235304

[B50] Tulchinsky D. Hobel C. J. (1973). Plasma human chorionic gonadotropin, estrone, estradiol, estriol, progesterone, and 17alpha-hydroxyprogesterone in human pregnancy. 3. Early normal pregnancy. *Am. J. Obstet. Gyn.* 117 884–893. 10.1016/0002-9378(73)90057-4 4759826

[B51] Tulchinsky D. Hobel C. J. Yeager E. Marshall J. R. (1972). Plasma estrone, estradiol, estriol, progesterone, and 17-hydroxyprogesterone in human pregnancy. I. Normal pregnancy. *Am. J. Obstet. Gyn.* 112 1095–1100. 10.1016/0002-9378(72)90185-8 5025870

[B52] Wang Y. Nan X. Zhao Y. Jiang L. Wang M. Wang H. (2021). Rumen microbiome structure and metabolites activity in dairy cows with clinical and subclinical mastitis. *J. Anim. Sci. Biotechnol.* 12:36. 10.1186/s40104-020-00543-1 33557959 PMC7869221

[B53] Wu G. D. Chen J. Hoffmann C. Bittinger K. Chen Y. Y. Keilbaugh S. A. (2011). Linking long-term dietary patterns with gut microbial enterotypes. *Science* 334 105–108. 21885731 10.1126/science.1208344 PMC3368382

[B54] Zeisel S. H. (1993). Choline phospholipids: signal transduction and carcinogenesis. *FASEB J.* 7 551–557. 8472893 10.1096/fasebj.7.6.8472893

[B55] Zhang R. Zhang J. Dang W. Irwin D. M. Zhang S. (2020). Unveiling the biogeography and potential functions of the intestinal digesta- and mucosa-associated microbiome of donkeys. *Front. Microbiol.* 11:596882. 10.3389/fmicb.2020.596882 33424800 PMC7793809

[B56] Zhang Z. W. Wang Y. H. Zhu M. X. Wang C. F. (2022). The in vitro digestion and fermentation characteristics of feedstuffs inoculated with cecal or colic fluid of Dezhou donkey. *J. Equine Vet. Sci.* 110:103864. 10.1016/j.jevs.2022.103864 35017038

[B57] Zhang Z. W. Zhan Y. D. Han Y. Liu Z. W. Wang Y. H. Wang C. F. (2021). Estimation of liveweight from body measurements through best fitted regression model in dezhou donkey breed. *J. Equine Vet. Sci.* 101:103457. 10.1016/j.jevs.2021.103457 33993924

