# Supplementary material for: Dynamic alterations in the donkey fecal bacteria community and metabolome characteristics during gestation
Source: Front Microbiol. 2022 Aug 18;13:927561. doi: 10.3389/fmicb.2022.927561 (PMC9434018; doi:10.3389/fmicb.2022.927561)
Supplement: Supplementary file 1 [file Data_Sheet_1.docx]

Supplementary Material

## Supplementary Figures

**Supplementary Figure 1.** Scatterplot from PCA shown the differences in the changes of donkey plasma metabolome during different gestation periods in the positive (a) and negative (b) ion mode. NP, no pregnant period; P1, pregnant period 1; P2, pregnant period 2; P3, pregnant period 3.

**Supplementary Figure 2.** KEGG pathway classification: metabolites detected and annotated. The x-axis represents the number of metabolites identified and the y-axis represents level-2 terms of the KEGG pathway.

## Supplementary Tables

**Table S1.** Ingredients and nutrient level of the basal diet for Dezhou donkeys.

| Items | Basal Diet |
| --- | --- |
| Ingredients (g/kg, as fed basis) |  |
| Wheat straw | 700 |
| Concentrate | 295 |
| Salt | 5 |
| Nutrient level 1 |  |
| Dry matter, g/kg (as fed basis) | 951.9 |
| Organic matter, g/kg DM | 915.3 |
| Crud protein, g/kg DM | 95.3 |
| Ether extract, g/kg DM | 17.7 |
| Neutral detergent fiber, g/kg DM | 566.3 |
| Acid detergent fiber, g/kg DM | 326.6 |

^1^ The nutrient level is the calculated values.

**Table S2.** Identification of differential metabolites in donkey plasma within different gestation periods.

| Metabolite | NP | P1 | P2 | P3 | P-value |
| --- | --- | --- | --- | --- | --- |
| Testolic acid | 0.51±0.54 | 3.70±0.88 | 3.84±2.00 | 5.13±0.15 | 1.6E-07 |
| 4R-Hydroxy solifenacin | 1.83±0.87 | 4.23±0.95 | 3.81±1.29 | 2.34±1.20 | 5.3E-03 |
| 14-Hydroxyandrosta-1,4-diene-3,17-dione | 2.16±0.02 | 3.17±0.8 | 3.65±1.28 | 4.22±0.19 | 3.8E-08 |
| 11-Oxo-androsterone glucuronide | 2.22±0.12 | 3.41±1.07 | 4.23±1.18 | 4.62±0.99 | 6.4E-04 |
| Cis-4-Hydroxyequol | 2.48±0.02 | 3.69±0.16 | 3.38±0.26 | 3.01±0.45 | 2.9E-07 |
| Equol 7-O-glucuronide | 2.74±0.02 | 4.03±0.30 | 2.93±0.27 | 2.90±0.25 | 4.9E-05 |
| Estradiol | 2.92±0.01 | 4.83±0.97 | 4.70±1.42 | 5.31±0.22 | 3.0E-08 |
| Oryzalide A | 2.97±0.01 | 3.37±0.76 | 4.16±1.08 | 5.00±0.26 | 6.3E-07 |
| Oryzalide B | 3.02±0.14 | 5.82±0.87 | 5.46±1.18 | 5.40±0.15 | 9.4E-10 |
| Lucidenic acid J | 3.12±0.73 | 4.11±0.75 | 4.25±0.61 | 4.84±0.30 | 2.8E-03 |
| Estrone 3-glucuronide | 3.18±0.01 | 3.84±0.82 | 4.64±0.98 | 5.47±0.34 | 1.5E-06 |
| Equol 4'-O-glucuronide | 3.22±0.04 | 4.44±0.26 | 3.47±0.29 | 3.47±0.27 | 2.5E-05 |
| Estrone | 3.33±0.01 | 3.57±0.64 | 4.17±0.99 | 4.88±0.25 | 3.6E-06 |
| Panaquinquecol 1 | 3.40±0.01 | 4.53±0.63 | 4.17±0.66 | 3.61±0.40 | 7.1E-03 |
| 4-Gingerol | 3.40±0.16 | 3.59±0.08 | 3.72±0.16 | 4.48±0.29 | 1.3E-04 |
| Tizoxanide glucuronide | 3.47±0.01 | 3.92±0.46 | 4.17±0.79 | 4.44±0.40 | 1.6E-03 |
| Taurochenodeoxycholate-7-sulfate | 3.58±0.48 | 3.53±0.47 | 4.08±0.27 | 4.51±0.20 | 1.3E-03 |
| Pregnanetriolone | 3.60±0.54 | 5.47±0.57 | 5.36±0.72 | 5.62±0.30 | 1.3E-04 |
| Estradiol-17beta 3-sulfate | 3.68±0.55 | 6.66±0.69 | 6.37±0.96 | 6.18±0.16 | 2.6E-05 |
| 2-Hydroxyestrone sulfate | 3.73±0.01 | 4.69±0.56 | 4.83±0.86 | 5.42±0.15 | 2.4E-08 |
| Deoxycholic acid 3-glucuronide | 3.83±0.14 | 4.62±0.37 | 4.74±0.50 | 5.25±0.34 | 2.1E-05 |
| Ethyl (S)-3-hydroxybutyrate glucoside | 3.89±0.44 | 3.62±0.43 | 4.16±0.72 | 3.14±0.19 | 5.9E-03 |
| Indolylacryloylglycine | 3.99±0.07 | 4.15±0.16 | 4.29±0.06 | 4.30±0.13 | 5.6E-05 |
| 4-Hydroxystachydrine | 4.02±0.40 | 3.79±0.35 | 4.43±0.51 | 2.92±0.27 | 1.3E-04 |
| Corchorusoside A | 4.05±0.39 | 4.85±0.59 | 4.96±0.31 | 5.69±0.27 | 6.3E-05 |
| 3-Hydroxytetradecanedioic acid | 4.18±0.25 | 3.91±0.07 | 4.12±0.15 | 2.89±0.31 | 7.4E-05 |
| Agecorynin A | 4.21±0.30 | 3.28±0.62 | 4.30±0.51 | 3.10±0.36 | 6.8E-04 |
| Histidinyl-Gamma-glutamate | 4.34±0.10 | 3.25±0.16 | 4.44±0.11 | 4.02±0.11 | 9.2E-08 |
| 2-Ethylacrylylcarnitine | 4.35±0.10 | 4.19±0.22 | 4.56±0.17 | 3.80±0.08 | 1.4E-06 |
| 16-Oxoestrone | 4.38±0.36 | 4.96±0.18 | 5.73±0.44 | 5.55±0.51 | 1.1E-03 |
| 6-{3,5-dihydroxy-2-[2-hydroxy-3-(3-hydroxyphenyl)propyl]phenoxy}-3,4,5-trihydroxyoxane-2-carboxylic acid | 4.42±0.39 | 5.06±0.11 | 4.85±0.15 | 4.62±0.21 | 2.1E-03 |
| N-Despropyl-rotigotine | 4.42±0.29 | 4.13±0.28 | 4.72±0.47 | 3.22±0.43 | 7.0E-04 |
| (1'R)-Nepetalic acid | 4.55±0.10 | 4.42±0.19 | 4.77±0.17 | 4.02±0.08 | 2.1E-06 |
| Pyrocatechol | 4.57±0.11 | 4.91±0.10 | 4.70±0.11 | 4.68±0.11 | 1.5E-03 |
| S-aminomethyldihydrolipoamide | 4.60±0.14 | 4.62±0.09 | 4.68±0.08 | 3.96±0.12 | 1.4E-06 |
| 3-amino-2-naphthoic acid | 4.63±0.23 | 4.96±0.34 | 5.35±0.33 | 5.48±0.48 | 3.6E-03 |
| 2,4-Nonadien-1-ol | 4.65±0.07 | 4.53±0.05 | 4.78±0.11 | 4.50±0.06 | 6.4E-04 |
| Chrysin 7-glucuronide | 4.66±0.15 | 5.08±0.09 | 4.77±0.10 | 4.12±0.16 | 1.4E-06 |
| Butyl (S)-3-hydroxybutyrate glucoside | 4.73±0.13 | 4.42±0.21 | 4.95±0.32 | 3.56±0.39 | 2.0E-04 |
| P-Anisic acid | 4.79±0.15 | 4.81±0.14 | 5.07±0.09 | 4.97±0.17 | 5.6E-03 |
| 8,11-Heptadecadienal | 4.79±0.15 | 4.78±0.09 | 4.88±0.10 | 4.56±0.10 | 1.5E-03 |
| 3-[3,5-dihydroxy-4-(sulfooxy)phenyl]propanoic acid | 4.86±0.09 | 4.90±0.07 | 5.01±0.06 | 4.75±0.12 | 4.3E-03 |
| 11'-Carboxy-alpha-chromanol | 4.88±0.15 | 4.96±0.21 | 4.92±0.21 | 5.23±0.11 | 4.2E-03 |
| (3a,5b,7a)-23-Carboxy-7-hydroxy-24-norcholan-3-yl-b-D-Glucopyranosiduronic acid | 4.90±0.30 | 5.74±0.37 | 5.71±0.41 | 6.27±0.23 | 4.7E-05 |
| Riesling acetal | 4.90±0.19 | 4.85±0.11 | 5.12±0.16 | 4.56±0.15 | 1.0E-03 |
| (+)-15,16-Dihydroxyoctadecanoic acid | 4.92±0.05 | 4.95±0.07 | 5.03±0.06 | 4.85±0.07 | 5.6E-03 |
| 3-(3,5-dihydroxyphenyl)-1-propanoic acid sulphate | 4.96±0.19 | 5.28±0.11 | 4.93±0.13 | 4.83±0.14 | 4.1E-04 |
| Cis-3-Chloroacrylic acid | 5.00±0.02 | 4.9±0.050 | 4.89±0.11 | 4.86±0.09 | 2.4E-03 |
| 5-Acetylamino-6-formylamino-3-methyluracil | 5.06±0.08 | 5.43±0.21 | 5.07±0.13 | 5.33±0.15 | 3.6E-03 |
| LysoPE(18:0/0:0) | 5.09±0.14 | 4.73±0.06 | 5.23±0.17 | 4.69±0.16 | 8.1E-05 |
| Suberic acid | 5.13±0.05 | 4.99±0.06 | 5.13±0.08 | 4.98±0.06 | 1.8E-03 |
| PE(P-16:0e/0:0) | 5.13±0.06 | 5.00±0.05 | 5.17±0.15 | 4.87±0.11 | 1.4E-03 |
| 2-Carboxy-4-dodecanolide | 5.15±0.08 | 5.16±0.09 | 5.31±0.10 | 4.95±0.15 | 5.4E-03 |
| LysoPC(20:4(5Z,8Z,11Z,14Z)) | 5.42±0.07 | 5.30±0.05 | 5.26±0.08 | 5.20±0.04 | 3.9E-04 |
| Uridine | 5.50±0.06 | 5.62±0.07 | 5.62±0.15 | 5.85±0.14 | 1.8E-03 |
| 2'',4'',6''-Triacetylglycitin | 5.50±0.05 | 5.42±0.02 | 5.49±0.04 | 5.44±0.05 | 4.8E-03 |
| 12-Oxo-2,3-dinor-10,15-phytodienoic acid | 5.55±0.07 | 5.43±0.02 | 5.51±0.04 | 5.46±0.03 | 3.5E-03 |
| 2,3-Dihydroxybenzoic acid | 5.55±0.07 | 5.60±0.08 | 5.72±0.06 | 5.54±0.06 | 1.7E-03 |
| (S)-10,16-Dihydroxyhexadecanoic acid | 5.58±0.09 | 5.71±0.16 | 5.90±0.09 | 5.74±0.22 | 1.3E-03 |
| 1,1'-[1,13-Tridecanediylbis(oxy)]bisbenzene | 5.58±0.04 | 5.47±0.02 | 5.51±0.03 | 5.56±0.04 | 4.9E-04 |
| (9R,10S,12Z)-9,10-Dihydroxy-8-oxo-12-octadecenoic acid | 5.67±0.09 | 5.65±0.10 | 5.78±0.13 | 5.45±0.08 | 7.2E-04 |
| Malathion monocarboxylic acid | 5.68±0.03 | 5.85±0.06 | 5.60±0.04 | 5.72±0.09 | 3.3E-05 |
| Avocadyne | 5.69±0.09 | 5.68±0.08 | 5.77±0.10 | 5.40±0.07 | 5.4E-05 |
| (2S,4R)-p-Mentha-1(7),5-dien-2-ol acetate | 5.77±0.04 | 5.69±0.02 | 5.73±0.03 | 5.70±0.03 | 4.0E-03 |
| Taurolithocholic acid 3-sulfate | 5.80±0.10 | 5.43±0.16 | 5.65±0.26 | 5.57±0.16 | 5.1E-03 |
| 15(S)-HpEDE | 5.83±0.10 | 5.9±0.070 | 5.85±0.08 | 5.65±0.09 | 3.3E-03 |
| Dimethylethanolamine | 5.88±0.03 | 5.85±0.01 | 5.86±0.01 | 5.85±0.01 | 2.0E-04 |
| 1,11-Undecanedicarboxylic acid | 5.88±0.07 | 5.88±0.12 | 6.02±0.05 | 5.71±0.12 | 1.3E-03 |
| MG(19:0/0:0/0:0) | 6.04±0.03 | 5.98±0.01 | 6.00±0.03 | 5.98±0.01 | 6.2E-03 |
| Pyrocatechol sulfate | 6.05±0.12 | 6.39±0.09 | 6.18±0.11 | 6.15±0.11 | 1.6E-03 |
| (+/-)13-HpODE | 6.22±0.08 | 6.15±0.05 | 6.27±0.10 | 5.99±0.09 | 1.5E-03 |
| Glutamylthreonine | 6.38±0.08 | 6.53±0.02 | 6.38±0.15 | 6.46±0.02 | 1.0E-03 |
| Hovenidulcigenin B | 6.44±0.05 | 6.32±0.04 | 6.33±0.07 | 6.32±0.05 | 3.6E-03 |
| L-Glutamine | 6.49±0.10 | 6.39±0.03 | 6.54±0.05 | 6.50±0.04 | 3.8E-04 |
| LysoPC(18:1(9Z)) | 6.71±0.04 | 6.59±0.04 | 6.65±0.10 | 6.54±0.07 | 7.6E-04 |
| LysoPC(18:2(9Z,12Z)) | 7.13±0.05 | 7.01±0.05 | 7.02±0.09 | 7.00±0.07 | 4.3E-03 |

NP, no pregnant period; P1, pregnant period 1; P2, pregnant period 2; P3, pregnant period 3.

**Table S3.** HMDB compound classification of differential metabolites in donkey plasma between NP and P1 groups.

| Metabolite | HMDB Subclass ^a^ | VIP | RT | Ino (M/Z) | FC (P1/NP) | P-value | mode |
| --- | --- | --- | --- | --- | --- | --- | --- |
| Testolic acid | - | 7.12 | 4.72 | 321.21 | 7.26 | 1.9E-05 | pos |
| (S)-Nerolidol 3-O-[a-L-rhamnopyranosyl-(1->2)-b-D-glucopyranoside] | Steroids and steroid derivatives | 4.11 | 6.14 | 511.29 | 2.94 | 4.6E-02 | neg |
| 4R-Hydroxy solifenacin | Prenol lipids | 4.29 | 4.40 | 415.14 | 2.32 | 1.0E-03 | neg |
| Oryzalide B | Organooxygen compounds | 4.87 | 6.29 | 319.19 | 1.93 | 1.4E-05 | neg |
| Estradiol-17beta 3-sulfate | Flavonoids | 5.05 | 6.21 | 351.13 | 1.81 | 9.0E-06 | neg |
| Estradiol | Isoflavonoids | 3.81 | 6.23 | 317.18 | 1.65 | 7.2E-04 | neg |
| 11-Oxo-androsterone glucuronide | Carboxylic acids and derivatives | 2.49 | 6.01 | 479.23 | 1.53 | 2.3E-02 | neg |
| Pregnanetriolone | Tetrahydroisoquinolines | 3.95 | 6.40 | 349.24 | 1.52 | 1.7E-04 | neg |
| Cis-4-Hydroxyequol | Fatty Acyls | 4.56 | 6.03 | 259.10 | 1.49 | 5.1E-09 | pos |
| Equol 7-O-glucuronide | Steroids and steroid derivatives | 4.64 | 5.26 | 436.16 | 1.47 | 9.6E-07 | pos |
| 14-Hydroxyandrosta-1,4-diene-3,17-dione | Steroids and steroid derivatives | 3.47 | 4.78 | 301.18 | 1.47 | 1.2E-02 | pos |
| 5,7-Megastigmadien-9-ol glucoside | - | 2.92 | 5.78 | 337.20 | 1.39 | 2.3E-03 | neg |
| Equol 4'-O-glucuronide | Steroids and steroid derivatives | 3.24 | 5.27 | 417.12 | 1.38 | 5.4E-07 | neg |
| Panaquinquecol 1 | - | 2.94 | 4.63 | 337.20 | 1.33 | 1.4E-03 | neg |
| Lucidenic acid J | - | 2.31 | 6.36 | 471.24 | 1.32 | 4.3E-02 | neg |
| 2-Hydroxyestrone sulfate | Organooxygen compounds | 2.61 | 6.19 | 365.11 | 1.26 | 1.8E-03 | neg |
| (1x,2x)-Guaiacylglycerol 3-glucoside | Fatty Acyls | 2.90 | 3.00 | 440.16 | 1.23 | 4.7E-02 | pos |
| Deoxycholic acid 3-glucuronide | Fatty Acyls | 3.39 | 6.38 | 586.36 | 1.21 | 6.6E-04 | pos |
| Threoninyl-Proline | Fatty Acyls | 2.03 | 3.55 | 237.09 | 1.20 | 3.8E-02 | neg |
| Corchorusoside A | Prenol lipids | 2.21 | 6.23 | 743.35 | 1.20 | 2.0E-02 | neg |
| (3a,5b,7a)-23-Carboxy-7-hydroxy-24-norcholan-3-yl-b-D-Glucopyranosiduronic acid | Purine nucleosides | 2.52 | 6.38 | 567.32 | 1.17 | 1.5E-03 | neg |
| 6-{3,5-dihydroxy-2-[2-hydroxy-3-(3-hydroxyphenyl)propyl]phenoxy}-3,4,5-trihydroxyoxane-2-carboxylic acid | Steroids and steroid derivatives | 2.10 | 5.93 | 433.11 | 1.15 | 3.0E-03 | neg |
| {3-[3-(2,5-dihydroxyphenyl)-3-oxopropyl]phenyl}oxidanesulfonic acid | Steroids and steroid derivatives | 1.72 | 6.04 | 337.04 | 1.13 | 3.3E-02 | neg |
| 16-Oxoestrone | - | 2.70 | 4.62 | 302.17 | 1.13 | 5.5E-03 | pos |
| Tizoxanide glucuronide | Steroids and steroid derivatives | 1.56 | 6.26 | 478.00 | 1.13 | 3.5E-02 | neg |
| Polyoxyethylene (600) monoricinoleate | Glycerolipids | 1.60 | 6.79 | 385.30 | 1.13 | 7.8E-03 | neg |
| 1-Methylhistidine | Stilbenes | 2.37 | 1.99 | 192.07 | 1.10 | 4.4E-03 | pos |
| METHIONAL | Carboxylic acids and derivatives | 2.38 | 4.33 | 105.04 | 1.09 | 8.6E-04 | pos |
| Chrysin 7-glucuronide | Organooxygen compounds | 2.54 | 4.19 | 431.10 | 1.09 | 1.9E-04 | pos |
| 6-({2-[(acetyloxy)methyl]-4,5,6-trihydroxyoxan-3-yl}oxy)-3,4,5-trihydroxyoxane-2-carboxylic acid | Carboxylic acids and derivatives | 1.74 | 3.75 | 379.09 | 1.09 | 5.9E-03 | neg |
| HEDERAGENIN | Pyridines and derivatives | 1.38 | 6.89 | 471.35 | 1.09 | 4.2E-02 | neg |
| Pyrocatechol | Prenol lipids | 1.66 | 3.02 | 109.03 | 1.07 | 2.2E-04 | neg |
| 5-Acetylamino-6-formylamino-3-methyluracil | Steroids and steroid derivatives | 1.65 | 3.23 | 271.07 | 1.07 | 2.4E-03 | neg |
| Tauroursodeoxycholic acid | - | 1.93 | 6.45 | 517.33 | 1.07 | 2.6E-02 | pos |
| Biliverdin | - | 1.87 | 6.38 | 583.25 | 1.06 | 1.9E-02 | pos |
| 3-(3,5-dihydroxyphenyl)-1-propanoic acid sulphate | Steroids and steroid derivatives | 1.47 | 3.33 | 261.01 | 1.06 | 5.1E-03 | neg |
| 2(3H)-Benzothiazolethione | Carboxylic acids and derivatives | 1.84 | 6.30 | 167.99 | 1.06 | 1.2E-02 | pos |
| 6-(3-ethenylphenoxy)-3,4,5-trihydroxyoxane-2-carboxylic acid | Steroids and steroid derivatives | 1.27 | 1.30 | 277.07 | 1.06 | 4.2E-02 | neg |
| Pyrocatechol sulfate | Fatty Acyls | 1.66 | 3.02 | 188.99 | 1.06 | 3.1E-04 | neg |
| 7-Methylguanosine 5'-phosphate | Tetrapyrroles and derivatives | 1.12 | 5.55 | 377.07 | 1.06 | 4.6E-02 | neg |
| Blighinone | Benzothiazoles | 1.50 | 1.72 | 366.99 | 1.06 | 2.3E-03 | neg |
| 4-Gingerol | - | 1.43 | 5.53 | 267.16 | 1.06 | 2.7E-02 | pos |
| [2-hydroxy-3-(7-hydroxy-4-oxo-4H-chromen-2-yl)-6-methoxyphenyl]oxidanesulfonic acid | Fatty Acyls | 2.12 | 1.04 | 418.98 | 1.06 | 2.4E-04 | pos |
| Ferulic acid 4-sulfate | - | 1.23 | 3.55 | 273.01 | 1.05 | 2.8E-02 | neg |
| Ethyl hydrogen fumarate | Glycerophospholipids | 1.26 | 2.06 | 189.04 | 1.05 | 9.9E-03 | neg |
| Armillarin | Glycerophospholipids | 1.21 | 6.59 | 459.21 | 1.05 | 2.3E-02 | neg |
| Ascorbic acid-2-sulfate | Carboxylic acids and derivatives | 1.13 | 1.17 | 254.98 | 1.05 | 4.3E-02 | neg |
| Tephrowatsin A | Carboxylic acids and derivatives | 1.58 | 6.18 | 355.19 | 1.05 | 3.4E-03 | pos |
| 3-methoxy Limaprost | Steroids and steroid derivatives | 1.26 | 6.79 | 411.28 | 1.05 | 6.3E-03 | neg |
| GPEtn(14:1/22:2) | Phenols | 1.69 | 7.46 | 742.54 | 1.04 | 3.9E-02 | pos |
| Xi-2,3-Dihydro-2-oxo-1H-indole-3-acetic acid | Organic sulfuric acids and derivatives | 1.55 | 4.39 | 233.09 | 1.04 | 3.2E-02 | pos |
| Indolylacryloylglycine | - | 1.34 | 4.16 | 227.08 | 1.04 | 4.0E-02 | pos |
| Val Ile | Diazines | 1.37 | 3.39 | 231.17 | 1.04 | 4.6E-02 | pos |
| Abiraterone sulfate | Fatty Acyls | 1.17 | 6.47 | 464.16 | 1.04 | 2.1E-02 | neg |
| 4-Hydroxybenzenesulfonic acid | - | 1.06 | 3.23 | 172.99 | 1.04 | 2.9E-02 | neg |
| 4alpha-Hydroxymethyl-4beta-methyl-5alpha-cholesta-8,24-dien-3beta-ol | Carboxylic acids and derivatives | 1.13 | 6.92 | 473.36 | 1.04 | 1.7E-02 | neg |
| 3-Methyl-L-histidine | Benzazepines | 1.68 | 1.16 | 170.09 | 1.04 | 7.8E-03 | pos |
| O-Acetylserine | Indoles and derivatives | 1.69 | 2.32 | 189.09 | 1.03 | 7.8E-04 | pos |
| Malathion monocarboxylic acid | Sphingolipids | 1.23 | 8.89 | 347.00 | 1.03 | 5.0E-05 | neg |
| Gamma-Glu-Leu | Phenanthrenes and derivatives | 1.04 | 3.55 | 259.13 | 1.03 | 2.2E-02 | neg |
| Menthyl ethylene glycol carbonate | Phenols | 1.06 | 6.60 | 487.33 | 1.03 | 1.2E-02 | neg |
| 4-Imidazolone-5-propionic acid | - | 1.34 | 2.84 | 189.09 | 1.03 | 7.8E-03 | pos |
| 3-Hydroxypicolinic acid | Organooxygen compounds | 1.17 | 2.60 | 140.03 | 1.02 | 2.6E-03 | pos |
| Glutamylthreonine | Carboxylic acids and derivatives | 1.02 | 1.10 | 247.09 | 1.02 | 2.4E-03 | neg |
| 5-Hydroxyindoleacetic acid | Prenol lipids | 1.24 | 2.82 | 192.07 | 1.02 | 4.1E-03 | pos |
| Dinorpromazine | - | 1.14 | 2.70 | 257.11 | 1.02 | 1.0E-02 | pos |
| Erythrono-1,4-lactone | Azoles | 1.24 | 1.12 | 160.06 | 1.02 | 6.0E-03 | pos |
| Glutamylglycine | Carboxylic acids and derivatives | 1.10 | 1.68 | 187.07 | 1.02 | 1.7E-02 | pos |
| Edetate | Peptidomimetics | 1.41 | 1.08 | 293.10 | 1.02 | 1.8E-03 | pos |
| L-beta-aspartyl-L-threonine | Indoles and derivatives | 1.28 | 1.20 | 235.09 | 1.02 | 1.7E-03 | pos |
| Porphobilinogen | - | 1.02 | 2.82 | 209.09 | 1.02 | 3.3E-02 | pos |
| N-Methyl-a-aminoisobutyric acid | Fatty Acyls | 1.06 | 1.24 | 118.09 | 0.99 | 1.2E-02 | pos |
| Acetyl-DL-Valine | - | 1.01 | 3.41 | 160.10 | 0.98 | 1.4E-03 | pos |
| L-Proline | Lactones | 1.08 | 1.07 | 116.07 | 0.98 | 3.2E-02 | pos |
| 2-Hydroxy-p-mentha-1,8-dien-6-one | Indoles and derivatives | 1.06 | 7.24 | 371.16 | 0.98 | 6.0E-03 | pos |
| L-Glutamine | Cinnamic acids and derivatives | 1.07 | 1.06 | 147.08 | 0.98 | 3.4E-02 | pos |
| Glycylproline | Fatty Acyls | 1.13 | 2.85 | 173.09 | 0.98 | 2.2E-02 | pos |
| 3-Formyl-6-hydroxyindole | Organooxygen compounds | 1.08 | 2.97 | 162.05 | 0.97 | 2.9E-02 | pos |
| Suberic acid | Steroids and steroid derivatives | 1.01 | 4.88 | 173.08 | 0.97 | 2.7E-03 | neg |
| 5,7-dihydroxy-2-(4-hydroxyphenyl)-8-(3,4,5-trihydroxyoxan-2-yl)-4H-chromen-4-one | Pyridines and derivatives | 1.04 | 6.13 | 401.09 | 0.97 | 8.5E-03 | neg |
| 2-Indolecarboxylic acid | - | 1.28 | 3.39 | 162.05 | 0.97 | 2.1E-03 | pos |
| LysoPC(18:3(6Z,9Z,12Z)) | Carboxylic acids and derivatives | 1.08 | 6.61 | 562.32 | 0.97 | 2.8E-03 | neg |
| 3,5,6-Trihydroxy-3',4',7-trimethoxyflavone 3-glucuronide | - | 1.11 | 6.13 | 517.10 | 0.97 | 8.5E-03 | neg |
| Asparaginyl-Proline | Prenol lipids | 1.56 | 6.52 | 459.22 | 0.96 | 6.6E-03 | pos |
| 1,4-Ipomeadiol | - | 1.03 | 5.19 | 385.19 | 0.96 | 4.0E-02 | neg |
| 3,4,5-trihydroxy-6-{[1-(4-methoxyphenyl)-4-methylpentan-3-yl]oxy}oxane-2-carboxylic acid | - | 1.02 | 5.62 | 383.17 | 0.96 | 3.9E-02 | neg |
| LysoSM(d18:0) | Carboxylic acids and derivatives | 1.51 | 6.64 | 484.38 | 0.96 | 3.2E-02 | pos |
| P-Cresol glucuronide | Glycerophospholipids | 1.41 | 4.51 | 283.08 | 0.94 | 9.7E-03 | neg |
| Acanthicifoline | Indoles and derivatives | 1.66 | 2.60 | 193.10 | 0.94 | 3.4E-02 | pos |
| Taurolithocholic acid 3-sulfate | Carboxylic acids and derivatives | 1.67 | 6.28 | 280.62 | 0.94 | 7.1E-04 | neg |
| 3-Hydroxytetradecanedioic acid | Carboxylic acids and derivatives | 1.74 | 6.09 | 297.17 | 0.94 | 2.9E-02 | pos |
| Butyl (S)-3-hydroxybutyrate glucoside | Prenol lipids | 1.90 | 3.85 | 287.15 | 0.94 | 1.2E-02 | pos |
| LysoPE(18:0/0:0) | Prenol lipids | 1.72 | 6.85 | 462.30 | 0.93 | 1.3E-04 | neg |
| Norendoxifen | - | 2.47 | 6.25 | 392.22 | 0.92 | 2.9E-03 | pos |
| 4-Hydroxyandrostenedione glucuronide | Carboxylic acids and derivatives | 2.03 | 2.70 | 511.25 | 0.91 | 4.0E-02 | pos |
| 3-Pyridinebutanoic acid | - | 2.35 | 3.29 | 207.11 | 0.90 | 2.6E-02 | pos |
| Adenosine | Carboxylic acids and derivatives | 2.74 | 2.60 | 268.10 | 0.90 | 2.1E-03 | pos |
| AzIII | Heteroaromatic compounds | 2.75 | 3.16 | 553.27 | 0.88 | 2.1E-03 | pos |
| Threoninyl-Lysine | Organonitrogen compounds | 2.46 | 3.50 | 212.14 | 0.87 | 3.8E-02 | pos |
| 2,8-Dihydroxyquinoline-beta-D-glucuronide | Carboxylic acids and derivatives | 3.13 | 3.65 | 338.09 | 0.80 | 2.4E-03 | pos |
| Agecorynin A | - | 3.37 | 3.46 | 389.12 | 0.78 | 8.1E-03 | pos |
| Histidinyl-Gamma-glutamate | Fatty Acyls | 4.31 | 2.78 | 284.13 | 0.75 | 5.2E-08 | pos |
| Kynuramine | - | 4.73 | 2.77 | 351.18 | 0.51 | 2.1E-02 | pos |

–: no pathway information; VIP, the contribution value of metabolites to the difference between the NP and P1 groups (VIP > 1); NP, no pregnant period; P1, pregnant period 1; FC, fold change, FC > 1 represents the upregulated compounds, while FC < 1 represents the downregulated compounds; P1/NP, P1 group vs. NP group; HMDB, Human metabolome database; VIP, Variable importance in the projection; RT, retention time; pos, positive ion mode; neg, negative ion mode.

**Table S4.** HMDB compound classification of differential metabolites in donkey plasma between NP and P2 groups.

| Metabolite | HMDB Subclass | VIP | RT | Ino (M/Z) | FC (P2/NP) | P-value | mode |
| --- | --- | --- | --- | --- | --- | --- | --- |
| Testolic acid | - | 8.31 | 4.72 | 321.21 | 7.53 | 0.003 | pos |
| 4R-Hydroxy solifenacin | Tetrahydroisoquinolines | 4.73 | 4.40 | 415.14 | 2.09 | 0.011 | neg |
| 11-Oxo-androsterone glucuronide | Glycerolipids | 5.04 | 6.01 | 479.23 | 1.90 | 0.002 | neg |
| Oryzalide B | Prenol lipids | 5.72 | 6.29 | 319.19 | 1.81 | 0.001 | neg |
| Estradiol-17beta 3-sulfate | Steroids and steroid derivatives | 6.16 | 6.21 | 351.13 | 1.73 | 0.000 | neg |
| 14-Hydroxyandrosta-1,4-diene-3,17-dione | - | 5.34 | 4.78 | 301.18 | 1.69 | 0.018 | pos |
| Estradiol | Steroids and steroid derivatives | 4.44 | 6.23 | 317.18 | 1.61 | 0.012 | neg |
| Pregnanetriolone | Steroids and steroid derivatives | 4.86 | 6.40 | 349.24 | 1.49 | 0.001 | neg |
| Estrone 3-glucuronide | Steroids and steroid derivatives | 4.17 | 6.13 | 445.19 | 1.46 | 0.005 | neg |
| Venoterpine | Pyridines and derivatives | 3.30 | 6.09 | 130.07 | 1.40 | 0.004 | neg |
| Oryzalide A | Prenol lipids | 3.50 | 6.05 | 319.19 | 1.40 | 0.023 | neg |
| Cis-4-Hydroxyequol | Isoflavonoids | 4.61 | 6.03 | 259.10 | 1.36 | 0.000 | pos |
| Lucidenic acid J | Prenol lipids | 3.49 | 6.36 | 471.24 | 1.36 | 0.015 | neg |
| 5,7-Megastigmadien-9-ol glucoside | Fatty Acyls | 3.22 | 5.78 | 337.20 | 1.35 | 0.023 | neg |
| 16-Oxoestrone | Steroids and steroid derivatives | 5.34 | 4.62 | 302.17 | 1.31 | 0.000 | pos |
| 2-Hydroxyestrone sulfate | Steroids and steroid derivatives | 3.51 | 6.19 | 365.11 | 1.29 | 0.011 | neg |
| Deoxycholic acid 3-glucuronide | Steroids and steroid derivatives | 4.43 | 6.38 | 586.36 | 1.24 | 0.002 | pos |
| 5-amino-2,5-dihydroxy-3,4-diphenylpentanoic acid | - | 3.18 | 6.07 | 284.13 | 1.23 | 0.046 | pos |
| Panaquinquecol 1 | Fatty Acyls | 2.86 | 4.63 | 337.20 | 1.23 | 0.018 | neg |
| Corchorusoside A | Steroids and steroid derivatives | 3.41 | 6.23 | 743.35 | 1.22 | 0.001 | neg |
| (3a,5b,7a)-23-Carboxy-7-hydroxy-24-norcholan-3-yl-b-D-Glucopyranosiduronic acid | Steroids and steroid derivatives | 3.16 | 6.38 | 567.32 | 1.17 | 0.003 | neg |
| 3-(3-Indolyl)-2-oxopropanoic acid | Indoles and derivatives | 3.09 | 4.69 | 204.07 | 1.16 | 0.016 | pos |
| 3-amino-2-naphthoic acid | - | 3.04 | 6.07 | 186.06 | 1.16 | 0.001 | neg |
| Taurochenodeoxycholate-7-sulfate | Steroids and steroid derivatives | 2.18 | 6.20 | 288.62 | 1.14 | 0.049 | neg |
| {3-[3-(2,5-dihydroxyphenyl)-3-oxopropyl]phenyl}oxidanesulfonic acid | - | 2.35 | 6.04 | 337.04 | 1.14 | 0.031 | neg |
| Kynuramine | Organooxygen compounds | 3.02 | 2.77 | 351.18 | 1.14 | 0.020 | pos |
| Indolelactic acid | Indoles and derivatives | 2.73 | 4.80 | 206.08 | 1.11 | 0.007 | pos |
| 2-Hydroxylinolenic acid | Fatty Acyls | 3.03 | 4.60 | 333.18 | 1.10 | 0.006 | pos |
| METHIONAL | Organooxygen compounds | 2.87 | 4.33 | 105.04 | 1.10 | 0.002 | pos |
| Tephrowatsin A | - | 2.78 | 6.18 | 355.19 | 1.10 | 0.005 | pos |
| 6-{3,5-dihydroxy-2-[2-hydroxy-3-(3-hydroxyphenyl)propyl]phenoxy}-3,4,5-trihydroxyoxane-2-carboxylic acid | - | 2.10 | 5.93 | 433.11 | 1.10 | 0.029 | neg |
| Indoleacrylic acid | Indoles and derivatives | 3.03 | 6.06 | 188.07 | 1.10 | 0.001 | pos |
| 4-Gingerol | - | 2.47 | 5.53 | 267.16 | 1.09 | 0.006 | pos |
| 3-Methylindole | Indoles and derivatives | 3.00 | 5.11 | 132.08 | 1.09 | 0.002 | pos |
| 7-Methylguanosine 5'-phosphate | - | 1.99 | 5.55 | 377.07 | 1.08 | 0.014 | neg |
| 2-(2,6-dihydroxy-3,4-dimethoxycyclohexylidene)acetonitrile | - | 2.70 | 5.11 | 178.09 | 1.08 | 0.005 | pos |
| Allyl butyrate | Fatty Acyls | 2.29 | 5.15 | 274.20 | 1.08 | 0.002 | pos |
| Indolylacryloylglycine | Carboxylic acids and derivatives | 2.76 | 4.16 | 227.08 | 1.08 | 0.000 | pos |
| 3-Indoleacetic Acid | Indoles and derivatives | 2.50 | 5.50 | 176.07 | 1.07 | 0.003 | pos |
| 2(3H)-Benzothiazolethione | Benzothiazoles | 2.39 | 6.30 | 167.99 | 1.07 | 0.012 | pos |
| Ascorbic acid-2-sulfate | - | 2.01 | 1.17 | 254.98 | 1.07 | 0.009 | neg |
| Ferulic acid 4-sulfate | Cinnamic acids and derivatives | 1.97 | 3.55 | 273.01 | 1.07 | 0.002 | neg |
| Xi-2,3-Dihydro-2-oxo-1H-indole-3-acetic acid | Indoles and derivatives | 2.55 | 4.39 | 233.09 | 1.07 | 0.004 | pos |
| Traumatic Acid | Fatty Acyls | 1.91 | 6.35 | 227.13 | 1.06 | 0.005 | neg |
| P-Anisic acid | Benzene and substituted derivatives | 1.89 | 6.21 | 151.04 | 1.06 | 0.002 | neg |
| Fluvoxamine acid | - | 1.88 | 5.46 | 319.13 | 1.06 | 0.029 | pos |
| Citpressine I | Quinolines and derivatives | 2.01 | 5.46 | 302.10 | 1.06 | 0.019 | pos |
| 4-Hydroxybenzenesulfonic acid | - | 1.88 | 3.23 | 172.99 | 1.06 | 0.012 | neg |
| (S)-10,16-Dihydroxyhexadecanoic acid | Fatty Acyls | 2.11 | 6.37 | 287.22 | 1.06 | 0.000 | neg |
| Indoleacetaldehyde | Indoles and derivatives | 2.33 | 2.77 | 160.08 | 1.06 | 0.017 | pos |
| 3-Methyl-quinolin-2-ol | - | 2.13 | 3.55 | 160.08 | 1.05 | 0.001 | pos |
| 2-Ethylacrylylcarnitine | Fatty Acyls | 1.97 | 5.55 | 244.15 | 1.05 | 0.021 | pos |
| (1'R)-Nepetalic acid | - | 2.00 | 5.57 | 226.14 | 1.05 | 0.019 | pos |
| 5-NITRO-2-PHENYLPROPYLAMINOBENZOIC ACID [NPPB] | - | 1.79 | 5.46 | 301.12 | 1.05 | 0.032 | pos |
| Succinylproline | - | 1.72 | 2.98 | 216.09 | 1.04 | 0.028 | pos |
| Fructose 1-phosphate | Organooxygen compounds | 1.49 | 3.45 | 296.98 | 1.04 | 0.011 | neg |
| Phenylacetylglycine methyl ester | - | 1.86 | 4.70 | 208.10 | 1.04 | 0.017 | pos |
| DICTAMNINE | Quinolines and derivatives | 1.83 | 5.57 | 200.07 | 1.04 | 0.012 | pos |
| 7-Methylinosine | Fatty Acyls | 1.77 | 4.27 | 266.10 | 1.04 | 0.020 | pos |
| 16,16-dimethyl-6-keto Prostaglandin E1 | - | 1.30 | 6.51 | 395.24 | 1.04 | 0.040 | neg |
| (+/-)-Enterolactone | Furanoid lignans | 1.86 | 5.53 | 299.13 | 1.04 | 0.021 | pos |
| 13,14-Dihydro PGF2a | Fatty Acyls | 1.59 | 6.38 | 355.25 | 1.04 | 0.003 | neg |
| 1,4'-Bipiperidine-1'-carboxylic acid | - | 1.32 | 6.28 | 423.30 | 1.03 | 0.034 | neg |
| 4-(2-Nitroethyl)phenyl primeveroside | Organooxygen compounds | 1.43 | 6.46 | 482.13 | 1.03 | 0.013 | neg |
| Armillarin | Organooxygen compounds | 1.28 | 6.59 | 459.21 | 1.03 | 0.042 | neg |
| 7-Hydroxycostol | Prenol lipids | 1.30 | 6.51 | 281.18 | 1.03 | 0.017 | neg |
| 5-Nonyltetrahydro-2-oxo-3-furancarboxylic acid | Lactones | 1.38 | 6.47 | 255.16 | 1.03 | 0.016 | neg |
| 2,2'-(3-methylcyclohexane-1,1-diyl)diacetic acid | - | 1.25 | 6.28 | 213.11 | 1.03 | 0.031 | neg |
| Ethyl hydrogen fumarate | Fatty Acyls | 1.21 | 2.06 | 189.04 | 1.03 | 0.038 | neg |
| 2-(3-Carboxy-3-(methylammonio)propyl)-L-histidine | Carboxylic acids and derivatives | 1.58 | 3.04 | 304.18 | 1.03 | 0.041 | pos |
| Cholesta-4,6-dien-3-one | Steroids and steroid derivatives | 1.63 | 6.40 | 424.36 | 1.03 | 0.032 | pos |
| 3-[3,5-dihydroxy-4-(sulfooxy)phenyl]propanoic acid | - | 1.30 | 6.56 | 277.00 | 1.03 | 0.009 | neg |
| 8-hydroxy-6,7-dimethoxy-2H-chromen-2-one | - | 1.32 | 6.35 | 203.03 | 1.03 | 0.009 | neg |
| 2-Carboxy-4-dodecanolide | Lactones | 1.30 | 6.41 | 241.14 | 1.03 | 0.013 | neg |
| Tiglylcarnitine | Fatty Acyls | 1.60 | 3.18 | 244.15 | 1.03 | 0.007 | pos |
| 2,3-Dihydroxybenzoic acid | Benzene and substituted derivatives | 1.48 | 4.05 | 153.02 | 1.03 | 0.001 | neg |
| 3-O-Methyl-L-DOPA | - | 1.61 | 2.75 | 212.09 | 1.03 | 0.003 | pos |
| C75 | - | 1.25 | 6.42 | 253.14 | 1.03 | 0.011 | neg |
| N-Acetyl-DL-methionine | - | 1.10 | 3.34 | 190.05 | 1.03 | 0.025 | neg |
| P-Menth-1-ene-8-thiol | Prenol lipids | 1.74 | 6.27 | 358.26 | 1.03 | 0.035 | pos |
| Enokipodin D | Prenol lipids | 1.63 | 3.62 | 280.15 | 1.03 | 0.025 | pos |
| 2,4-Nonadien-1-ol | - | 1.12 | 6.47 | 185.12 | 1.03 | 0.033 | neg |
| (R)-2-Hydroxysterculic acid | Fatty Acyls | 1.26 | 6.61 | 355.25 | 1.03 | 0.022 | neg |
| Pimelic acid | Fatty Acyls | 1.20 | 3.93 | 159.07 | 1.03 | 0.009 | neg |
| Isokobusone | Organooxygen compounds | 1.13 | 6.47 | 267.16 | 1.03 | 0.023 | neg |
| 13,14-dihydro Prostaglandin F1alpha | - | 1.35 | 6.44 | 357.27 | 1.02 | 0.009 | neg |
| Butyryl-L-carnitine | Fatty Acyls | 1.83 | 2.84 | 232.15 | 1.02 | 0.001 | pos |
| Antibiotic X 14889A | Prenol lipids | 1.61 | 6.61 | 648.46 | 1.02 | 0.004 | pos |
| 3,5,7-Trimethyl-2E,4E,6E,8E-decatetraene | - | 1.33 | 5.49 | 177.16 | 1.02 | 0.021 | pos |
| 1,11-Undecanedicarboxylic acid | Fatty Acyls | 1.27 | 6.47 | 243.16 | 1.02 | 0.004 | neg |
| 2(5H)-Furanone | Dihydrofurans | 1.36 | 2.84 | 85.03 | 1.02 | 0.011 | pos |
| Dopamine quinone | Organooxygen compounds | 1.46 | 3.44 | 134.06 | 1.02 | 0.020 | pos |
| Indoxylsulfuric acid | Organic sulfuric acids and derivatives | 1.15 | 3.45 | 212.00 | 1.02 | 0.039 | neg |
| (+)-15,16-Dihydroxyoctadecanoic acid | Fatty Acyls | 1.10 | 6.62 | 315.25 | 1.02 | 0.008 | neg |
| 5-Hydroxyindoleacetic acid | Indoles and derivatives | 1.38 | 2.82 | 192.07 | 1.02 | 0.015 | pos |
| Dinorpromazine | - | 1.44 | 2.70 | 257.11 | 1.02 | 0.013 | pos |
| N2-Acetyl-L-ornithine | Carboxylic acids and derivatives | 1.15 | 1.14 | 173.09 | 1.02 | 0.007 | neg |
| Menthyl ethylene glycol carbonate | Prenol lipids | 1.10 | 6.60 | 487.33 | 1.02 | 0.030 | neg |
| L-2-Amino-3-methylenehexanoic acid | Carboxylic acids and derivatives | 1.71 | 1.09 | 144.10 | 1.02 | 0.001 | pos |
| Porphobilinogen | Organonitrogen compounds | 1.34 | 2.82 | 209.09 | 1.02 | 0.030 | pos |
| Pro Pro | - | 1.21 | 2.57 | 213.12 | 1.02 | 0.022 | pos |
| Cis-p-Menthane-1,7,8-triol | Prenol lipids | 1.11 | 6.49 | 375.28 | 1.02 | 0.027 | neg |
| Trimethylamine N-oxide | Organonitrogen compounds | 1.41 | 0.99 | 76.08 | 1.02 | 0.033 | pos |
| Isoleucyl-Lysine | Carboxylic acids and derivatives | 1.36 | 6.63 | 560.42 | 1.02 | 0.009 | pos |
| PE(18:1/0:0) | - | 1.38 | 6.80 | 502.29 | 1.02 | 0.011 | pos |
| 3-hydroxypristanic acid | Prenol lipids | 1.09 | 6.59 | 359.28 | 1.02 | 0.026 | neg |
| Creatinine | Carboxylic acids and derivatives | 1.31 | 1.03 | 227.12 | 1.02 | 0.029 | pos |
| Polyoxyethylene 40 monostearate | Fatty Acyls | 1.01 | 6.44 | 346.33 | 1.01 | 0.034 | pos |
| Agavoside A | Steroids and steroid derivatives | 1.11 | 6.94 | 631.33 | 0.99 | 0.029 | pos |
| PC(18:2/0:0) | - | 1.24 | 6.69 | 520.34 | 0.99 | 0.018 | pos |
| Malathion monocarboxylic acid | Fatty Acyls | 1.00 | 8.89 | 347.00 | 0.99 | 0.002 | neg |
| LysoPC(18:2(9Z,12Z)) | Glycerophospholipids | 1.11 | 6.69 | 564.33 | 0.98 | 0.016 | neg |
| Hovenidulcigenin B | Prenol lipids | 1.09 | 6.68 | 581.32 | 0.98 | 0.013 | neg |
| 2-Hydroxy-p-mentha-1,8-dien-6-one | Prenol lipids | 1.31 | 7.24 | 371.16 | 0.98 | 0.003 | pos |
| Linoleic acid | Fatty Acyls | 1.01 | 6.70 | 279.23 | 0.98 | 0.025 | neg |
| Mammea B/BC cyclo E | Coumarins and derivatives | 1.20 | 7.02 | 355.16 | 0.98 | 0.027 | neg |
| Palmitoyl-L-carnitine | Fatty Acyls | 1.47 | 6.52 | 400.34 | 0.97 | 0.012 | pos |
| (+/-)-Myristoylcarnitine | - | 1.36 | 6.45 | 372.31 | 0.97 | 0.044 | pos |
| Asparaginyl-Proline | Carboxylic acids and derivatives | 1.51 | 6.52 | 459.22 | 0.97 | 0.031 | pos |
| LysoPC(20:4(5Z,8Z,11Z,14Z)) | Glycerophospholipids | 1.41 | 6.67 | 588.33 | 0.97 | 0.003 | neg |
| Sterebin A | Prenol lipids | 1.78 | 6.30 | 328.25 | 0.96 | 0.028 | pos |
| 4-Hydroxyandrostenedione glucuronide | Steroids and steroid derivatives | 1.96 | 2.70 | 511.25 | 0.95 | 0.043 | pos |
| Norendoxifen | Stilbenes | 2.46 | 6.25 | 392.22 | 0.94 | 0.003 | pos |
| Ala Ile Glu His | - | 2.08 | 2.62 | 469.24 | 0.91 | 0.045 | pos |

–: no pathway information; VIP, the contribution value of metabolites to the difference between the NP and P2 groups (VIP > 1); NP, no pregnant period; P2, pregnant period 2; FC, fold change, FC > 1 represents the upregulated compounds, while FC < 1 represents the downregulated compounds; P2/NP, P2 group vs. NP group; HMDB, Human metabolome database; VIP, Variable importance in the projection; RT, retention time; pos, positive ion mode; neg, negative ion mode.

**Table S5.** HMDB compound classification of differential metabolites in donkey plasma between NP and P3 groups.

| Metabolite | HMDB Subclass | VIP | RT | Ino (M/Z) | FC (P3/NP) | P_value | mode |
| --- | --- | --- | --- | --- | --- | --- | --- |
| Testolic acid | - | 8.79 | 4.72 | 321.21 | 10.06 | 2.0E-09 | pos |
| Deoxycholic acid 3-glucuronide | Steroids and steroid derivatives | 4.78 | 6.01 | 479.23 | 2.08 | 1.5E-04 | neg |
| 14-Hydroxyandrosta-1,4-diene-3,17-dione | - | 5.86 | 4.78 | 301.18 | 1.95 | 1.7E-10 | pos |
| Estradiol | Steroids and steroid derivatives | 5.04 | 6.23 | 317.18 | 1.82 | 1.2E-10 | neg |
| Oryzalide B | Prenol lipids | 5.03 | 6.29 | 319.19 | 1.79 | 6.2E-11 | neg |
| Estrone 3-glucuronide | Steroids and steroid derivatives | 4.92 | 6.13 | 445.19 | 1.72 | 1.4E-08 | neg |
| Oryzalide A | Prenol lipids | 4.64 | 6.05 | 319.19 | 1.68 | 4.1E-09 | neg |
| Estradiol-17beta 3-sulfate | Steroids and steroid derivatives | 5.06 | 6.21 | 351.13 | 1.68 | 8.9E-07 | neg |
| Pregnanetriolone | Steroids and steroid derivatives | 4.50 | 6.40 | 349.24 | 1.56 | 1.1E-05 | neg |
| 16-Oxoestrone | Steroids and steroid derivatives | 3.90 | 6.36 | 471.24 | 1.55 | 3.3E-04 | neg |
| Estrone | Steroids and steroid derivatives | 4.04 | 6.26 | 315.16 | 1.47 | 3.3E-08 | neg |
| Butyl (S)-3-hydroxybutyrate glucoside | Fatty Acyls | 4.21 | 6.19 | 365.11 | 1.45 | 9.1E-11 | neg |
| Lucidenic acid D1 | Prenol lipids | 3.10 | 6.37 | 469.23 | 1.43 | 1.2E-02 | neg |
| 4-Hydroxystachydrine | Carboxylic acids and derivatives | 4.08 | 6.23 | 743.35 | 1.41 | 7.1E-06 | neg |
| Glycosides | Steroids and steroid derivatives | 2.32 | 6.09 | 130.07 | 1.37 | 3.5E-02 | neg |
| 11-Oxo-androsterone glucuronide | Glycerolipids | 4.83 | 6.38 | 586.36 | 1.37 | 3.0E-06 | pos |
| Corchorusoside A | Steroids and steroid derivatives | 4.06 | 5.53 | 267.16 | 1.32 | 1.1E-05 | pos |
| Neuromedin N (1-4) | Fatty Acyls | 2.93 | 6.07 | 284.13 | 1.31 | 3.8E-02 | pos |
| (3a,5b,7a)-23-Carboxy-7-hydroxy-24-norcholan-3-yl-b-D-Glucopyranosiduronic acid | Steroids and steroid derivatives | 3.75 | 6.38 | 567.32 | 1.28 | 4.6E-06 | neg |
| Adenosine | Purine nucleosides | 3.06 | 6.26 | 478.00 | 1.28 | 1.4E-04 | neg |
| Lucidenic acid J | Prenol lipids | 3.98 | 4.62 | 302.17 | 1.27 | 1.0E-03 | pos |
| AMRINONE | Pyridines and derivatives | 2.68 | 1.35 | 167.02 | 1.27 | 4.9E-02 | neg |
| 4-Carboxynevirapine | Steroids and steroid derivatives | 2.98 | 6.20 | 288.62 | 1.26 | 1.4E-03 | neg |
| Ethyl (S)-3-hydroxybutyrate glucoside | Fatty Acyls | 2.50 | 6.94 | 313.28 | 1.23 | 2.5E-03 | neg |
| Uric acid | Imidazopyrimidines | 2.51 | 6.03 | 259.10 | 1.21 | 1.7E-02 | pos |
| Topaquinone | Carboxylic acids and derivatives | 2.78 | 6.40 | 535.28 | 1.21 | 7.7E-03 | neg |
| 7-Aminoclonazepam | Benzodiazepines | 2.67 | 6.07 | 186.06 | 1.18 | 2.7E-03 | neg |
| Venoterpine | Pyridines and derivatives | 2.32 | 4.60 | 333.18 | 1.10 | 1.0E-02 | pos |
| Histidinyl-Gamma-glutamate | Carboxylic acids and derivatives | 2.14 | 5.11 | 132.08 | 1.10 | 2.7E-02 | pos |
| Indolylacryloylglycine | Carboxylic acids and derivatives | 2.16 | 5.11 | 178.09 | 1.10 | 2.8E-02 | pos |
| 3-Methylindole | Indoles and derivatives | 2.18 | 5.27 | 601.27 | 1.10 | 2.6E-02 | pos |
| Tryptophanamide | Indoles and derivatives | 1.95 | 4.80 | 206.08 | 1.09 | 2.0E-02 | pos |
| GPEtn(18:2/18:2) | Glycerophospholipids | 2.09 | 1.99 | 192.07 | 1.09 | 1.1E-02 | pos |
| 3-hydroxytetradecanoyl carnitine | Fatty Acyls | 1.98 | 4.33 | 105.04 | 1.09 | 1.8E-02 | pos |
| HEDERAGENIN | Prenol lipids | 1.62 | 6.89 | 471.35 | 1.08 | 1.4E-02 | neg |
| Uracil | Diazines | 1.60 | 6.33 | 289.02 | 1.08 | 1.4E-02 | neg |
| Aspartyl-Arginine | Carboxylic acids and derivatives | 2.20 | 4.16 | 227.08 | 1.08 | 3.0E-04 | pos |
| METHIONAL | Organooxygen compounds | 1.97 | 7.33 | 740.52 | 1.08 | 3.5E-02 | pos |
| Indoleacrylic acid | Indoles and derivatives | 1.96 | 6.06 | 188.07 | 1.08 | 1.1E-02 | pos |
| Citpressine I | Quinolines and derivatives | 1.73 | 6.61 | 463.31 | 1.07 | 9.3E-04 | neg |
| Fluvoxamine acid | - | 1.80 | 5.46 | 319.13 | 1.07 | 1.2E-02 | pos |
| Uridine | Pyrimidine nucleosides | 1.85 | 1.98 | 243.06 | 1.06 | 2.4E-04 | neg |
| 4-Hydroxyandrostenedione glucuronide | Steroids and steroid derivatives | 2.10 | 6.42 | 370.29 | 1.06 | 2.1E-02 | pos |
| Pro Tyr Gly | - | 1.79 | 5.46 | 302.10 | 1.06 | 1.3E-02 | pos |
| Riesling acetal | Tetrahydrofurans | 1.67 | 5.46 | 301.12 | 1.06 | 1.6E-02 | pos |
| Genipic acid | Dihydrofurans | 1.52 | 3.23 | 271.07 | 1.05 | 2.9E-03 | neg |
| (9R,10S,12Z)-9,10-Dihydroxy-8-oxo-12-octadecenoic acid | Fatty Acyls | 1.42 | 1.37 | 111.01 | 1.05 | 9.7E-03 | neg |
| 6,7-Dimethoxy-1-Methyl-3,4-Dihydroisoquinoline | - | 1.88 | 3.28 | 247.14 | 1.05 | 2.3E-02 | pos |
| 16,16-dimethyl-6-keto Prostaglandin E1 | - | 1.28 | 6.51 | 395.24 | 1.05 | 1.3E-02 | neg |
| 1-Heptadecanoylglycerophosphoethanolamine | Glycerophospholipids | 1.25 | 3.34 | 190.05 | 1.05 | 1.2E-02 | neg |
| 1-Palmitoyl-2-linoleoyl PE | - | 1.26 | 7.47 | 714.51 | 1.04 | 4.3E-02 | neg |
| [2-hydroxy-3-(7-hydroxy-4-oxo-4H-chromen-2-yl)-6-methoxyphenyl]oxidanesulfonic acid | - | 1.68 | 2.75 | 212.09 | 1.04 | 2.8E-04 | pos |
| LysoPC(20:4(5Z,8Z,11Z,14Z)) | Glycerophospholipids | 1.46 | 5.57 | 200.07 | 1.04 | 1.4E-02 | pos |
| 3-O-Methyl-L-DOPA | - | 1.69 | 1.04 | 418.98 | 1.04 | 2.8E-03 | pos |
| 3-Methyl-L-histidine | Carboxylic acids and derivatives | 1.78 | 1.16 | 170.09 | 1.04 | 5.9E-03 | pos |
| PC(14:0/0:0) | Glycerophospholipids | 1.54 | 2.91 | 202.11 | 1.04 | 8.5E-03 | pos |
| [1-(2-hydroxyphenyl)-2-oxo-2-phenylethoxy]sulfonic acid | - | 1.57 | 2.18 | 113.03 | 1.04 | 5.6E-03 | pos |
| 2-Carboxy-4-dodecanolide | Lactones | 1.19 | 6.92 | 473.36 | 1.04 | 2.5E-02 | neg |
| LysoPC(18:2(9Z,12Z)) | Glycerophospholipids | 1.05 | 6.35 | 203.03 | 1.03 | 1.4E-02 | neg |
| Suberic acid | Fatty Acyls | 1.16 | 2.57 | 213.12 | 1.03 | 2.3E-02 | pos |
| Phosphocholine | Organonitrogen compounds | 1.14 | 1.37 | 191.02 | 1.03 | 1.7E-02 | neg |
| 8,11-Heptadecadienal | Fatty Acyls | 1.32 | 1.68 | 187.07 | 1.03 | 1.2E-02 | pos |
| 2,3-Dinor-TXB2 | Fatty Acyls | 1.26 | 1.65 | 210.06 | 1.03 | 4.4E-02 | pos |
| 4-Pyrimidine Methanamine (hydrochloride) | - | 1.12 | 1.06 | 110.07 | 1.03 | 2.7E-02 | pos |
| L-2-Amino-3-methylenehexanoic acid | Carboxylic acids and derivatives | 1.02 | 3.30 | 181.05 | 1.02 | 7.6E-03 | neg |
| 4alpha-Hydroxymethyl-4beta-methyl-5alpha-cholesta-8,24-dien-3beta-ol | Prenol lipids | 1.19 | 1.03 | 227.12 | 1.02 | 4.6E-02 | pos |
| Pro Pro | - | 1.16 | 2.70 | 257.11 | 1.02 | 4.8E-03 | pos |
| PC(16:0/0:0)[U] | - | 1.18 | 2.32 | 189.09 | 1.02 | 1.0E-02 | pos |
| PC(18:2/0:0) | - | 1.01 | 1.09 | 144.10 | 1.01 | 1.0E-02 | pos |
| (Z)-3-(1-Formyl-1-propenyl)pentanedioic acid | Fatty Acyls | 1.01 | 6.69 | 520.34 | 0.99 | 4.5E-02 | pos |
| LysoPC(14:0/0:0) | Glycerophospholipids | 1.19 | 6.77 | 496.34 | 0.98 | 2.0E-02 | pos |
| Agavoside A | Steroids and steroid derivatives | 1.24 | 6.94 | 631.33 | 0.98 | 8.2E-04 | pos |
| 2,4-Nonadien-1-ol | - | 1.15 | 6.77 | 459.25 | 0.98 | 5.7E-03 | pos |
| LysoPC(15:0) | Glycerophospholipids | 1.09 | 1.06 | 146.12 | 0.98 | 3.3E-02 | pos |
| 8-hydroxy-6,7-dimethoxy-2H-chromen-2-one | - | 1.05 | 6.69 | 564.33 | 0.98 | 2.4E-03 | neg |
| Hovenidulcigenin B | Prenol lipids | 1.04 | 6.68 | 581.32 | 0.98 | 1.4E-03 | neg |
| O-Acetylserine | Carboxylic acids and derivatives | 1.20 | 6.84 | 510.35 | 0.98 | 3.0E-02 | pos |
| Pyridoxine (Vitamin B6) | - | 1.16 | 6.77 | 184.07 | 0.98 | 1.7E-02 | pos |
| Cis-3-Chloroacrylic acid | Carboxylic acids and derivatives | 1.09 | 6.77 | 480.31 | 0.98 | 2.3E-03 | neg |
| (+/-)13-HpODE | - | 1.48 | 6.78 | 522.35 | 0.98 | 3.6E-03 | pos |
| Creatinine | Carboxylic acids and derivatives | 1.18 | 2.91 | 170.08 | 0.98 | 2.1E-02 | pos |
| Isocitrate | Carboxylic acids and derivatives | 1.18 | 6.79 | 566.35 | 0.98 | 5.7E-04 | neg |
| LysoPE(0:0/18:0) | Glycerophospholipids | 1.22 | 6.72 | 546.36 | 0.98 | 2.2E-02 | pos |
| 3-Formyl-6-hydroxyindole | Indoles and derivatives | 1.33 | 2.85 | 173.09 | 0.97 | 1.2E-02 | pos |
| Glutamylglycine | Carboxylic acids and derivatives | 1.35 | 6.70 | 482.32 | 0.97 | 3.0E-02 | pos |
| Acetylcholine | Organonitrogen compounds | 1.10 | 6.70 | 316.94 | 0.97 | 3.0E-03 | neg |
| 1,11-Undecanedicarboxylic acid | Fatty Acyls | 1.10 | 6.47 | 243.16 | 0.97 | 1.8E-02 | neg |
| Linoleic acid | Fatty Acyls | 1.14 | 6.70 | 279.23 | 0.97 | 3.8E-03 | neg |
| 15(S)-HpEDE | - | 1.18 | 4.88 | 173.08 | 0.97 | 7.6E-04 | neg |
| 1-heptadecanoyl-sn-glycero-3-phosphocholine | - | 1.23 | 6.73 | 339.25 | 0.97 | 1.0E-02 | neg |
| Gly Pro Val | - | 1.59 | 6.64 | 468.31 | 0.97 | 1.2E-02 | pos |
| (2Z)-6-hydroxy-2-(phenylmethylidene)heptanal | - | 1.14 | 6.47 | 185.12 | 0.97 | 2.6E-03 | neg |
| Glycylproline | Carboxylic acids and derivatives | 1.33 | 5.48 | 387.20 | 0.97 | 1.2E-02 | neg |
| PC(15:0/0:0) | Glycerophospholipids | 1.37 | 2.97 | 162.05 | 0.97 | 1.1E-02 | pos |
| LysoPC(18:1(9Z)) | Glycerophospholipids | 1.20 | 6.71 | 526.32 | 0.97 | 6.4E-03 | neg |
| 1-(8Z,11Z,14Z-eicosatrienoyl)-sn-glycero-3-phosphocholine | - | 1.23 | 6.64 | 512.30 | 0.96 | 8.3E-03 | neg |
| DICTAMNINE | Quinolines and derivatives | 1.48 | 6.50 | 311.22 | 0.96 | 6.3E-04 | neg |
| N-Acetyl-DL-methionine | - | 1.26 | 6.41 | 241.14 | 0.96 | 1.8E-02 | neg |
| 3-Furoic acid | Furans | 1.38 | 6.40 | 327.22 | 0.96 | 1.0E-03 | neg |
| D-Glucaro-1,4-lactone | Lactones | 1.24 | 6.28 | 280.62 | 0.96 | 1.4E-02 | neg |
| LysoPC(18:1(11Z)) | Glycerophospholipids | 1.43 | 6.67 | 588.33 | 0.96 | 4.3E-05 | neg |
| Dinorpromazine | - | 1.23 | 6.71 | 466.29 | 0.96 | 3.5E-02 | neg |
| 3a,7a,12b-Trihydroxy-5b-cholanoic acid | - | 1.55 | 2.82 | 272.16 | 0.95 | 4.0E-02 | pos |
| Taurolithocholic acid 3-sulfate | Steroids and steroid derivatives | 1.25 | 6.64 | 295.23 | 0.95 | 1.0E-02 | neg |
| Acanthicifoline | - | 1.58 | 2.60 | 193.10 | 0.95 | 2.2E-02 | pos |
| Avocadyne | Fatty Acyls | 1.66 | 6.44 | 329.23 | 0.95 | 9.5E-05 | neg |
| 5-Acetylamino-6-formylamino-3-methyluracil | Diazines | 1.48 | 6.84 | 436.28 | 0.95 | 4.1E-04 | neg |
| 11'-Carboxy-alpha-chromanol | - | 1.76 | 2.91 | 336.15 | 0.95 | 1.8E-02 | pos |
| PE(P-16:0e/0:0) | - | 1.52 | 6.54 | 426.32 | 0.95 | 3.8E-02 | pos |
| Deoxycholic acid glycine conjugate | Steroids and steroid derivatives | 1.54 | 6.51 | 448.31 | 0.94 | 2.5E-02 | neg |
| LysoPE(0:0/20:1(11Z)) | Glycerophospholipids | 1.52 | 6.73 | 552.33 | 0.94 | 8.6E-03 | neg |
| LysoPE(18:0/0:0) | Glycerophospholipids | 1.86 | 2.82 | 329.18 | 0.93 | 4.0E-02 | pos |
| 5-NITRO-2-PHENYLPROPYLAMINOBENZOIC ACID [NPPB] | - | 1.66 | 6.19 | 271.16 | 0.93 | 6.4E-03 | neg |
| Indolelactic acid | Indoles and derivatives | 1.91 | 2.68 | 470.22 | 0.93 | 3.6E-02 | pos |
| 2-(2,6-dihydroxy-3,4-dimethoxycyclohexylidene)acetonitrile | - | 2.13 | 2.78 | 284.13 | 0.93 | 3.1E-04 | pos |
| 1-Methylhistidine | Carboxylic acids and derivatives | 2.11 | 2.70 | 511.25 | 0.92 | 2.3E-02 | pos |
| Gly Gly Pro Val | - | 1.91 | 6.85 | 462.30 | 0.92 | 8.3E-04 | neg |
| Cis-and trans-5-Ethyl-4-methyl-2-(2-butyl)-thiazoline | - | 2.36 | 6.42 | 412.28 | 0.90 | 4.5E-02 | pos |
| 2-Hydroxylinolenic acid | Fatty Acyls | 2.34 | 3.26 | 549.26 | 0.90 | 3.4E-02 | pos |
| 5-amino-2,5-dihydroxy-3,4-diphenylpentanoic acid | - | 2.82 | 5.57 | 226.14 | 0.88 | 1.6E-06 | pos |
| Chrysin 7-glucuronide | - | 2.88 | 4.19 | 431.10 | 0.88 | 1.6E-04 | pos |
| Tizoxanide glucuronide | Benzazepines | 3.07 | 2.60 | 268.10 | 0.88 | 1.1E-03 | pos |
| 2-Ethylacrylylcarnitine | Fatty Acyls | 3.01 | 5.55 | 244.15 | 0.87 | 8.1E-07 | pos |
| S-aminomethyldihydrolipoamide | Fatty Acyls | 3.20 | 5.78 | 269.14 | 0.86 | 6.0E-06 | pos |
| 3-amino-2-naphthoic acid | - | 2.66 | 2.78 | 188.08 | 0.86 | 2.2E-02 | pos |
| Taurochenodeoxycholate-7-sulfate | Steroids and steroid derivatives | 2.87 | 3.27 | 527.28 | 0.81 | 4.2E-02 | pos |
| Cis-4-Hydroxyequol | Isoflavonoids | 2.43 | 3.30 | 275.11 | 0.81 | 3.4E-03 | neg |
| (1'R)-Nepetalic acid | - | 2.92 | 2.98 | 297.10 | 0.79 | 4.4E-02 | pos |
| N-Despropyl-rotigotine | Tetralins | 4.24 | 3.85 | 287.15 | 0.75 | 3.3E-05 | pos |
| Tuftsin | Carboxylic acids and derivatives | 2.75 | 6.24 | 192.03 | 0.74 | 2.0E-02 | neg |
| Stearaldehyde | Fatty Acyls | 2.51 | 6.00 | 284.06 | 0.74 | 2.7E-02 | neg |
| 4-Gingerol | - | 4.10 | 3.46 | 389.12 | 0.74 | 1.8E-04 | pos |
| 2-Hydroxyestrone sulfate | Steroids and steroid derivatives | 4.25 | 3.44 | 274.13 | 0.73 | 2.0E-04 | pos |
| Agecorynin A | - | 4.11 | 3.44 | 319.19 | 0.73 | 2.1E-04 | pos |
| 3-Hydroxytetradecanedioic acid | Fatty Acyls | 4.52 | 6.09 | 297.17 | 0.69 | 1.2E-05 | pos |

–: no pathway information; VIP, the contribution value of metabolites to the difference between the NP and P3 groups (VIP > 1); NP, no pregnant period; P3, pregnant period 2; FC, fold change, FC > 1 represents the upregulated compounds, while FC < 1 represents the downregulated compounds; P3/NP, P3 group vs. NP group; HMDB, Human metabolome database; VIP, Variable importance in the projection; RT, retention time; pos, positive ion mode; neg, negative ion mode.
